# Empirical comparison of health-related quality of life and subjective well-being measures in Australian adolescents

**DOI:** 10.1007/s11136-026-04331-8

**Published:** 2026-07-04

**Authors:** Kaung Mon Winn, Maame Esi Woode, Gang Chen

**Affiliations:** 1 Centre for Health Economics, Monash University, Caulfield Campus, Caulfield East, Melbourne, VIC 3145 Australia; 2 Cancer Health Services Research, University of Melbourne, Melbourne, VIC 3000 Australia; 3 Peter MacCallum Cancer Centre, Melbourne, VIC 3000 Australia

**Keywords:** Health-related quality of life, Subjective well-being, CHU9D, EQ-5D-5L, DWI

## Abstract

**Background:**

Health-related quality of life (HRQoL) and subjective well-being (SWB) are increasingly used to capture aspects of welfare beyond traditional health indicators; however, their conceptual distinction has not been fully resolved.

**Objective:**

This study examines the empirical relationships between HRQoL, measured using Child Health Utility 9D (CHU9D), EQ-5D-5L and EQ-5D-5L with psychosocial bolt-ons and SWB, measured using a 12-item Life Satisfaction Scale for Youth (LSS-Y) and the Disability Wellbeing Index (DWI), among Australian adolescents, and explores factors associated with these outcomes.

**Methods:**

A nationwide, quota-based online survey was administered to Australian adolescents aged 15–19 years. The survey included the above instruments and sociodemographic questions. Psychometric analyses, including Spearman’s correlation, Kruskal–Wallis tests, and exploratory factor analyses (EFA), were conducted to examine interrelationships and latent structures. Regression analyses were used to identify factors associated with the outcome measures.

**Results:**

Data were collected from 1026 adolescents (44.5% female). Of these, 510 completed the CHU9D and 516 completed the EQ-5D-5L with psychosocial bolt-ons under a randomised design; all participants completed the LSS-Y and DWI. No HRQoL dimension showed strong correlations (Spearman’s *ρ* > 0.60) with SWB dimensions, and EFA revealed limited overlap in latent constructs. Regression analyses identified general health, socioeconomic status, gender, migration status, and disability status as significant determinants of adolescents’ health and well-being.

**Conclusion:**

HRQoL and SWB are not interchangeable but provide complementary insights. Using both measures may offer a more comprehensive understanding of adolescents’ overall well-being.

**Supplementary Information:**

The online version contains supplementary material available at https://doi.org/10.1007/s11136-026-04331-8.

## Introduction

Health-related quality of life (HRQoL) has long been the dominant outcome construct in health economics and policy evaluation. Preference-based HRQoL measures, known as multi-attribute utility instruments (MAUIs), classify health states and assign preference-based utility scores to derive quality-adjusted life years (QALYs) [1]. These measures provide a standardised and comparable framework for capturing health status, and have been widely adopted across clinical areas and population groups, including children and adolescents. As a result, HRQoL remains central to economic evaluation and decision-making in health systems internationally.

However, HRQoL is a subset of quality of life (QoL), primarily applied in health and medical research, where QoL is inherently broad and multidimensional. The World Health Organization (WHO) defines QoL as “individuals’ perception of their position in life in the context of the culture and value systems in which they live and in relation to their goals, expectations, standards and concerns” [2] (p. 1405). In economic and policy evaluation, outcome measurement aims to assess the overall impact of interventions to inform resource allocation and decision-making. Consequently, many scholars argue that health alone, as captured by QALYs, may not fully reflect the total impact of policies or programs on individuals’ lives, highlighting the need to consider broader aspects of well-being beyond HRQoL [3]. In response, there has been increasing interest in measuring well-being, particularly through subjective well-being (SWB), which can capture broader life aspects extending beyond health [4, 5]. SWB reflects how individuals evaluate their own lives, offering insights into personal experiences and values and serving as a useful indicator of socioeconomic and societal progress [6, 7]. Its multidimensional scope provides a broader conceptual foundation for outcome measurement than health-focused frameworks alone.

Several preference-based HRQoL measures have been developed for young people [8], differing in their descriptive systems and valuation approaches. For example, EQ-5D instruments primarily focus on physical health, whereas the Child Health Utility 9D (CHU9D) places greater emphasis on psychosocial functioning relevant to younger populations [9].

Similarly, several well-being instruments have also been developed, including a small number of preference-based measures [10]. One example is Investigating Choice Experiments CAPability (ICECAP) Measures, originally developed for adults, grounded in Sen’s capability approach, which defines well-being as the ability to *do* and *be* the things one values [11, 12]. Another example is the 25-item EQ Health and Wellbeing instrument, which was developed for adults and designed to capture a broad range of health and well-being dimensions; its 9-item short form (EQ-HWB-9) can be used to derive preference-based scores for calculating QALYs in economic evaluations [13, 14]. A third example is the 14-item Disability Wellbeing Index (DWI), developed to assess the SWB of people with disabilities (aged 15 years and above) [15]. The DWI scoring algorithm is currently available only for people with disabilities, ICECAP and EQ-HWB-9 are restricted to adults, and the development of ICECAP for children and young people remains in its early stages [16].

This raises an important empirical question: do well-being measures provide additional information beyond HRQoL instruments regarding individuals’ overall life circumstances and the broader impact of interventions? Studies comparing ICECAP-A (for adults) and ICECAP-O (for older populations) with EQ-5D instruments reported correlations of *ρ*= 0.49 and *ρ*= 0.47, respectively, indicating partial overlap between capability well-being and health-focused measures [17, 18]. Chen et al. [19] further found that ICECAP-A correlated more strongly with AQoL-8D (*ρ*= 0.802), which incorporates broader psychosocial domains, than with EQ-5D-5L (*ρ*= 0.604), suggesting that the degree of overlap depends on the conceptual breadth of the comparator instrument. Evidence from younger populations similarly indicates that EQ-5D-Y demonstrates low to moderate correlations with KIDSCREEN instruments—multidimensional child QoL measures encompassing psychological and social domains—but stronger associations with psychosocial subscales [20]. Collectively, these findings suggest that HRQoL instruments primarily focused on physical health may not fully capture broader aspects of well-being related to social and psychological life circumstances.

To the best of our knowledge, no empirical study has compared HRQoL and SWB measures in adolescent populations. Adolescence is defined by the WHO as 10–19 years, and the Lancet Commission further situates this period within a broader developmental phase spanning 10–24 years, distinguishing early adolescence as 10–14 years, late adolescence as 15–19 years, and young adulthood as 20–24 years [21]. Adolescence is a critical developmental period, as well-being during this stage strongly influences later adult outcomes including health [22–24]. Given their ongoing development and age-specific experiences, adolescents’ well-being is shaped by developmental and contextual factors that vary across the life course [25–27]. This highlights the need for research and policy to recognise these distinct factors and incorporate adolescent-specific well-being measures that capture broader aspects of their lived experience.

This study examined the empirical relationships between HRQoL and SWB measures among Australian adolescents using three HRQoL instruments: the CHU9D, the EQ-5D-5L, and the EQ-5D-5L with psychosocial bolt-on dimensions, and two SWB instruments: a study-specific 12-item life-domain satisfaction question set (hereafter referred to as Life Satisfaction Scale for Youth (LSS-Y); see Methods for details), and the 14-item DWI. Specifically, this study addressed the following research questions:

1. To what extent do HRQoL and SWB measures demonstrate empirical agreement in adolescent populations?
2. Do HRQoL and SWB measures exhibit structural overlap or distinct dimensionality?
3. Do these instruments differentiate adolescents by general health status and socioeconomic status?
4. How are HRQoL and SWB scores associated with sociodemographic characteristics in adjusted analyses?

## Methods

### Sample and survey

Data for this study were obtained from a nationwide, quota-based online survey of Australian adolescents aged 15–19 years, administered through the Student Edge Youth Panel (https://youthpanel.studentedge.org). The survey comprised four sections. The first section collected participants’ background information, including three basic demographic characteristics (age, gender, and state of residence) for quota screening purposes, and their satisfaction with life domains using the LSS-Y. The second section included discrete choice questions to examine the relative importance of life domains (reported elsewhere). The third section gathered SWB and HRQoL data using DWI [15] and CHU9D [28] or EQ-5D-5L plus Psychosocial bolt-ons [5]. The last section captured additional sociodemographic characteristics, including self-reported general health status, socioeconomic status at both national and school levels, education status, disability status, migration status, language spoken at home, loneliness, and expectations about the future.

The survey was designed to collect data from a broadly nationally representative cohort of Australian adolescents to examine and compare HRQoL and SWB. Quotas were developed based on age, gender, and state of residence using population distributions from the Australian Bureau of Statistics, and recruitment was monitored to ensure that these quota targets were met. We included DWI in our study to measure the SWB of adolescents, even though it was developed for use in adolescents with disabilities, because it is a multidimensional SWB measure for adolescents, and its items can apply to the general adolescent population. However, the study did not aim to oversample adolescents with disabilities; rather, all measures were administered to the general adolescent population.

 The study was approved by the Monash University Human Research Ethics Committee, Monash University, Australia (Project ID: 39889).

#### Instrument description

*Life Satisfaction Scale for Youth (LSS-Y)*: LSS-Y is a study-specific set of life-domain satisfaction questions, assessing cognitive well-being across youth-relevant life domains. The question set was introduced with the stem “*How satisfied are you with each of the following?*”, followed by twelve life domains: *physical health*,* mental health*,* appearance*,* life at school*,* friends*,* neighbourhood*,* things you have*,* time use*,* family*,* future*, and *choice*. These life domains were drawn from children and adolescent-specific SWB and quality-of-life instruments, informed by a systematic review of self-reported multidimensional measures for young people, further comparison with well-established youth well-being instruments, and refined through consultation with adolescents to assess relevance and age-appropriate wording (see Online Resource 1 for details). Responses were recorded on four ordered evaluative categories (Excellent, Good, Fair, Poor), which have been used in similar well-being instruments (e.g. the Quality of Life Enjoyment and Satisfaction Questionnaire) [29] and were further refined through adolescent consultation to enhance clarity and interpretability, rendering 4^12^ = 16,777,216 possible well-being states. 

*Disability Wellbeing Index (DWI)*: DWI is a preference-based well-being instrument comprising two components: the first captures how respondents *feel* about various aspects of their lives, and the second optional component assesses how *important* those aspects are to them. The scoring algorithm, derived from the first component, was developed using data from Australian adults and young people with disabilities aged 15 years and above [15]. The instrument covers fourteen items: *family*,* friendships*,* people who support me*,* personal care*,* everyday activities*,* meaningful life*,* housing*,* physical health*,* mental health*,* safety*,* learning*,* respect and dignity*,* finances*, and *work*. Each item has five response levels (1 = all of the time to 5 = none of the time), yielding 5^14^ = 6,103,515,625 possible well-being states.

The currently available DWI preference weights were derived from populations with disabilities and were therefore not applied to the present general adolescent sample. Preference weights reflect the normative value structure of the population from which they are elicited; applying disability-specific weights to a different population may limit comparability. Moreover, the preference-weighted LSS-Y scoring algorithm is still under development. Accordingly, in this study, non-preference-weighted approaches were used to generate summative scores for the SWB measures.

In line with the DWI scoring approach [15] and established discussions on aggregation in multidimensional well-being measurement [30–32], two types of non-preference-weighted mean scores were calculated: *arithmetic (compensatory)* and *harmonic (non-compensatory)* means. The arithmetic mean assumes full compensability across domains, whereas the harmonic mean places greater weight on lower domain scores and reduces the extent to which high achievement in one domain can offset deficits in another. In multidimensional well-being measurement, non-compensatory aggregation has been argued to better reflect situations in which severe disadvantage in one domain cannot be fully substituted by strengths in others [30, 31]. Therefore, we used harmonic mean scores in regression analyses as the primary non-compensatory summary scores.

The arithmetic and harmonic mean scores for each individual were calculated using the following formulas:

$$\:\boldsymbol{Arithmetic}\:\boldsymbol{Mean}=\sum_{i=1}^{N}\frac{{x}_{i}}{N}$$

$$\:\boldsymbol{Harmonic}\:\boldsymbol{Mean}=\frac{N}{\sum\nolimits_{i=1}^{N}\frac{1}{{x}_{i}}}$$

where* i* denotes each dimension (life domain or aspect),$\:{x}_{i}$ represents the raw score of dimension *i*, and * N* is the number of items. All the mean scores were rescaled to a 0–1 range using the following equation, with higher scores indicating better well-being.

$$\:Scores\:\left(rescaled\right)=\frac{{Score}_{Maximum}-{Score}_{Mean}}{{Score}_{Maximum}-{Score}_{Minimum}}$$

where ${Score}_{Mean}$ is the arithmetic or harmonic mean score, and and ${Score}_{Minimum}$ present to the maximum and minimum scores of the instruments in theory. 

*Child Health Utility 9D (CHU9D)*: CHU9D was developed and validated to measure HRQoL in children aged 7–17 years [33–35]. It has nine dimensions: *worried*,* sad*,* pain*,* tired*,* annoyed*,* schoolwork*,* sleep*,* daily routine*, and *activities.* Each dimension has five response levels, generating 5^9^ = 1,953,125 possible health states. CHU9D utility scores were calculated using the Australian adolescent-specific preference-based algorithm [28]. 

*EQ-5D-5L*: EQ-5D-5L is a preference-based measure developed by the EuroQol Group to assess HRQoL in individuals aged ≥ 15 years [36, 37]. It comprises five dimensions: *mobility*,* self-care*,* usual activities*,* pain/discomfort*, and *anxiety/depression*. Each has five response levels, producing 5^5^ = 3125 possible health states. 

*Psychosocial Bolt-on* *dimensions* *for* *EQ-5D-5L*: Four additional dimensions, including *vitality*,* sleep*,* social relationships*, and *community connectedness*, were proposed to extend the EQ-5D-5L, aimed at addressing the absence of psychosocial aspects, by Chen and Olsen [5]. Every dimension has five response levels, resulting in 5^9^ = 1,953,125 possible health states. In this study, respondents completed all nine items. Responses to all nine items were scored using the mapping algorithm developed by Chen and Olsen [5], while the value set reported by Norman et al. [38] was applied to the original five EQ-5D-5L dimensions only to derive the standard EQ-5D-5L utility scores. Both algorithms reflect Australian population preferences.

Table 1 provides a descriptive summary of the instruments included in the study.

**Table 1:** Descriptive summary of included outcome measures

	Life satisfaction scale for youth	Disability wellbeing index	CHU9D	EQ-5D-5L	EQ-5D-5L + Psychosocial Bolt-ons
Descriptive system					
Conceptual type	Subjective well-being	Subjective well-being	Health-related quality of life	Health-related quality of life	Health-related quality of life
Targeted populations	Adolescents aged 15–19 years	People with disability, including young people with disability	Children and adolescents aged 7–17 years	General population, including young people	General population
No. of Dimensions	12	14	9	5	9
No. of Items	12	14	9	5	9
Response levels	4	5	5	5	5
Well-being or health states defined	16,777,216	6,103,515,625	1,953,125	3125	1,953,125
Recall period	Not specified*	Current	Today	Today	Today & Last week
Scoring system					
Types of scoring	Summative score	Summative score	Utility score	Utility score	Utility score
Score range (Minimum–Maximum)	0–1	0–1	(− 0.106) − 1 (Australian Adolescents)	(− 0.301) − 1 (Australian General Population)	0.047–1 (Australian General Population)
Dimensions included	Mental health; Physical health; Family; Friends; Life at school; Neighbourhood; Time use; Safety; Things you have; Appearance; Future; Choice	Mental health; Physical health; Family; Friendships; Learning; Housing; Everyday activities; Safety; Meaningful life; Personal care; Respect and dignity; People who support me; Finances; Work	Worried; Annoyed; Sad; Sleep; Tired Pain; Schoolwork / homework; Daily routine; Activities	Anxiety / depression; Mobility; Pain / discomfort; Usual activities; Self-care	Anxiety / depression; Mobility; Pain / discomfort; Usual activities; Self-care; Sleep; Vitality; Community connectedness; Social relationships

CHU9D = Child Health Utility 9D; *LSS-Y does not specify a recall period, consistent with the OECD Well-Being Questionnaire for the Programme for International Student Assessment (PISA) and Children’s Worlds: The International Survey of Children’s Well-Being, which also do not apply a recall period to life-domain satisfaction questions

### Statistical analysis

Descriptive analyses were conducted by calculating the mean (± Standard Deviation, SD) and median (with interquartile range, IQR) scores of the outcome measures across sociodemographic groups.

To address Research Question 1, Spearman’s correlation coefficients were computed to assess the strength of associations between the dimensions of the SWB and HRQoL instruments. Correlation strength (*ρ*) was interpreted as follows: 0–0.19 very weak, 0.20–0.39 weak, 0.40–0.59 moderate, 0.60–0.79 strong, and 0.80–1.00 very strong [39]. It was hypothesised that overall correlations between SWB and HRQoL measures would be low, reflecting their complementary rather than substitutable roles. However, stronger correlations were expected between health-related domains of SWB—particularly mental health—and corresponding psychosocial dimensions of HRQoL, compared with physical health domains.

To address Research Question 2, exploratory factor analysis (EFA) was conducted on pooled items from each SWB and HRQoL instrument to investigate the underlying dimensional structure and content overlap between pairs of instruments. Data suitability was assessed by Kaiser–Meyer–Olkin (KMO) measure and Bartlett’s test of sphericity. KMO values between 0.80 and 1.00 indicate adequate sampling, while a significant Bartlett’s test (*p* < 0.05) suggests sufficient correlations among variables for factor analysis. EFA was performed using the maximum likelihood method. The number of factors was determined using the minimum average partial method, shown to outperform alternative approaches [19, 40, 41], and supported by parallel analysis. To explore a range of plausible factor structures, models with two to four factors were estimated and evaluated for interpretability. Model selection was guided by fit indices, including the Root Mean Square Error of Approximation (RMSEA), Akaike Information Criterion (AIC), Bayesian Information Criterion (BIC), Comparative Fit Index (CFI), and Tucker–Lewis Index (TLI). Given the expected correlations among factors, oblique (Promax) rotation was applied. Items with factor loadings below 0.30 were suppressed and excluded from interpretation [19].

To address Research Question 3, known-groups validity was assessed by testing whether the instruments could discriminate between adolescents differing in self-reported general health and socioeconomic status (SES), which are theoretically and empirically associated with variations in HRQoL and SWB in adolescent populations [35, 42–44]. General health status was measured as an ordinal variable with five levels (poor, fair, good, very good, and excellent). SES was assessed using the youth version of the MacArthur Scale of Subjective Social Status, a 10-point ladder scale (1–10) [45], which provides two ratings: one reflecting perceived family standing in Australian society (national level), and one reflecting perceived standing within the school community (school level). For interpretability and to ensure adequate group sizes, SES was recategorised into three groups: low (1–4), middle (5–7), and high (8–10). Normality of outcome data was evaluated using the Shapiro–Francia test, which rejected the null hypothesis of normality (*p* < 0.05) for all instruments. Consequently, the non-parametric Kruskal–Wallis test was used to compare the scores across groups defined by self-reported general health and SES. Known-groups validity was further examined using multivariable regression analyses after adjusting for sociodemographic covariates.

Finally, to address Research Question 4, linear regression analyses were conducted to examine the associations between sociodemographic characteristics and adolescents’ health and well-being. The scores of the outcome measures were regressed on key sociodemographic variables, including age group, gender, educational status (≤ secondary and tertiary level education), self-reported general health, disability status and SES. Disability status was measured as a self-reported binary indicator (yes/no), defined by the presence of any impairment or long-term health condition with self-identification as having a disability.

All statistical analyses were performed using STATA version 18 [46], except for the Spearman’s correlations and EFA, which were conducted using EViews version 14 [47].

## Results

### Descriptive atatistics

A total of 2330 adolescent online panel members were sent the survey questionnaire link. Of these, 1304 participants were excluded for being outside the target age range (*N* = 311), not providing consent (*N* = 704), submitting incomplete responses to the survey or well-being measures (*N* = 270), and completing the entire survey too quickly (i.e., in less than 3 min) (*N* = 19), which may indicate low-quality responses. After exclusions, 1026 participants remained in the final sample: 510 completed the CHU9D and 516 completed the EQ-5D-5L with psychosocial bolt-ons, as each respondent was randomly assigned one HRQoL instrument. Online Resource 2 presents participant flow to the final sample and subsamples.

The mean age of respondents was 17 years, with 44.5% identifying as female. Over 70% were born in Australia, and fewer than 30% were attending university. Table 2 presents descriptive statistics (means and standard deviations) of scores of the outcome measures stratified by sociodemographic characteristics. Corresponding medians and interquartile ranges of the scores of the outcome measures are provided in Online Resource 2.

### Agreement between SWB and HRQoL measures

None of the HRQoL dimensions showed strong correlations (*ρ* ≥ 0.60) with any domains of the SWB measures. Correlation coefficients ranged from − 0.029 to 0.595 for LSS-Y–HRQoL and from 0.049 to 0.564 for DWI–HRQoL comparisons, indicating predominantly weak to moderate associations. Detailed correlation matrices are presented in Online Resource 3.

For the LSS-Y, the *mental health* domain showed the highest correlations with HRQoL dimensions. Among the CHU9D domains, all correlations with LSS-Y domains were weak, except for a moderate correlation between the *sad* domain and the LSS-Y *mental health* domain (*ρ* = 0.488). Within the EQ-5D-5L, the *anxiety* domain had the strongest correlation with the LSS-Y *mental health* domain (*ρ* = 0.595, moderate-to-high), and a moderate correlation with the *life at school* domain (*ρ* = 0.483), while the remaining EQ-5D-5L dimensions showed only weak correlations with LSS-Y domains. The psychosocial bolt-on dimensions showed moderate correlations with LSS-Y *mental health* (ranging from 0.437 to 0.526), whereas correlations with other LSS-Y domains were generally weak to moderate.

For the DWI, the *mental health* dimension similarly demonstrated the strongest correlations with HRQoL domains. The CHU9D *sad* domain and the EQ-5D-5L *anxiety* domain showed moderate correlations with DWI *mental health* (*ρ* = 0.467 and 0.564, respectively). The psychosocial bolt-on dimensions were moderately correlated with DWI *mental health* (*ρ* = 0.419–0.500), while correlations between HRQoL physical domains and other DWI domains were weak to moderate.

**Table 2:** Characteristics of participants along with the means and standard deviations of outcome scores

Participant Characteristics	*N* or Mean (% or SD) – Full Sample	Life Satisfaction Scale for Youth (LSS-Y)		Disability Wellbeing Index (DWI)		*N* or Mean (% or SD) – CHU9D Sample	CHU9D	*N* or Mean (% or SD) – EQ-5D Sample	EQ-5D-5L	EQ-5D-5 L with Psychosocial Bolt-ons
		Harmonic Mean Scores	Arithmetic Mean Scores	Harmonic Mean Scores	Arithmetic Mean Scores		Utility Scores		Utility Scores	
Mean ± SD		Mean ± SD	Mean ± SD	Mean ± SD	Mean ± SD		Mean ± SD		Mean ± SD	Mean ± SD
Sample size	1026 (100.00)	0.692 ± 0.170	0.615 ± 0.177	0.756 ± 0.149	0.693 ± 0.159	510 (49.71)	0.539 ± 0.239	516 (50.29)	0.886 ± 0.161	0.663 ± 0.197
Age groups										
15 years	170 (16.57)	0.708 ± 0.170	0.637 ± 0.175	0.777 ± 0.149	0.717 ± 0.163	84 (16.47)	0.550 ± 0.253	86 (16.67)	0.901 ± 0.159	0.550 ± 0.253
16 years	213 (20.76)	0.694 ± 0.170	0.618 ± 0.178	0.758 ± 0.152	0.692 ± 0.165	109 (21.37)	0.497 ± 0.250	104 (20.16)	0.906 ± 0.141	0.497 ± 0.250
17 years	209 (20.37)	0.690 ± 0.173	0.614 ± 0.178	0.754 ± 0.147	0.693 ± 0.156	98 (19.22)	0.541 ± 0.241	111 (21.51)	0.873 ± 0.183	0.541 ± 0.241
18 years	231 (22.51)	0.684 ± 0.164	0.603 ± 0.169	0.754 ± 0.138	0.687 ± 0.149	119 (23.33)	0.563 ± 0.216	112 (21.71)	0.886 ± 0.145	0.563 ± 0.216
19 years	203 (19.79)	0.686 ± 0.174	0.609 ± 0.184	0.741 ± 0.158	0.679 ± 0.165	100 (19.61)	0.547 ± 0.239	103 (19.96)	0.865 ± 0.170	0.547 ± 0.239
Gender										
Girl	457 (44.54)	0.658 ± 0.156	0.579 ± 0.157	0.736 ± 0.146	0.672 ± 0.153	229 (44.90)	0.495 ± 0.235	228 (44.19)	0.878 ± 0.159	0.623 ± 0.181
Boy	548 (53.41)	0.721 ± 0.175	0.646 ± 0.186	0.774 ± 0.149	0.711 ± 0.163	271 (53.14)	0.576 ± 0.236	277 (53.68)	0.891 ± 0.163	0.699 ± 0.204
Unknown / Others	21 (2.05)	0.675 ± 0.183	0.593 ± 0.195	0.734 ± 0.157	0.664 ± 0.164	10 (1.96)	0.564 ± 0.253	11 (2.13)	0.907 ± 0.114	0.597 ± 0.191
States										
Australian Capital Territory	21 (2.05)	0.713 ± 0.214	0.656 ± 0.230	0.790 ± 0.145	0.736 ± 0.155	9 (1.76)	0.448 ± 0.229	12 (2.33)	0.941 ± 0.081	0.766 ± 0.189
South Australia	71 (6.92)	0.672 ± 0.168	0.591 ± 0.173	0.730 ± 0.154	0.661 ± 0.162	42 (8.24)	0.471 ± 0.234	29 (5.62)	0.883 ± 0.161	0.658 ± 0.188
New South Wales	389 (37.91)	0.707 ± 0.166	0.631 ± 0.174	0.769 ± 0.145	0.706 ± 0.157	193 (37.84)	0.561 ± 0.240	196 (37.98)	0.901 ± 0.150	0.680 ± 0.196
Tasmania	11 (1.07)	0.551 ± 0.189	0.497 ± 0.180	0.676 ± 0.127	0.622 ± 0.135	8 (1.57)	0.433 ± 0.229	3 (0.58)	0.908 ± 0.133	0.569 ± 0.181
Northern Territory	5 (0.49)	0.675 ± 0.071	0.606 ± 0.087	0.582 ± 0.133	0.507 ± 0.140	2 (0.39)	0.500 ± 0.051	3 (0.58)	0.753 ± 0.157	0.462 ± 0.087
Victoria	282 (27.49)	0.686 ± 0.179	0.611 ± 0.185	0.753 ± 0.157	0.693 ± 0.167	133 (26.08)	0.546 ± 0.237	149 (28.88)	0.875 ± 0.153	0.650 ± 0.201
Queensland	120 (11.70)	0.683 ± 0.146	0.600 ± 0.153	0.734 ± 0.140	0.663 ± 0.148	53 (10.39)	0.524 ± 0.231	67 (12.98)	0.895 ± 0.129	0.652 ± 0.170
Western Australia	127 (12.38)	0.685 ± 0.172	0.610 ± 0.178	0.768 ± 0.144	0.703 ± 0.151	70 (13.73)	0.546 ± 0.250	57 (11.05)	0.842 ± 0.238	0.649 ± 0.224
Australian Born										
No	268 (26.12)	0.712 ± 0.166	0.638 ± 0.174	0.779 ± 0.139	0.715 ± 0.150	137 (26.86)	0.599 ± 0.231	131 (25.39)	0.899 ± 0.157	0.599 ± 0.231
Yes	758 (73.88)	0.685 ± 0.171	0.607 ± 0.177	0.748 ± 0.152	0.685 ± 0.162	373 (73.14)	0.518 ± 0.239	385 (74.61)	0.881 ± 0.162	0.518 ± 0.239
Education										
≤ Secondary education	760 (74.07)	0.693 ± 0.171	0.616 ± 0.178	0.760 ± 0.151	0.695 ± 0.163	370 (72.55)	0.535 ± 0.241	390 (75.58)	0.886 ± 0.163	0.670 ± 0.197
≥ Tertiary education	266 (25.93)	0.687 ± 0.166	0.612 ± 0.172	0.747 ± 0.143	0.686 ± 0.150	140 (27.45)	0.550 ± 0.234	126 (24.42)	0.885 ± 0.154	0.643 ± 0.197
General health										
Poor	36 (3.51)	0.472 ± 0.194	0.372 ± 0.185	0.565 ± 0.171	0.477 ± 0.164	18 (3.53)	0.233 ± 0.141	18 (3.49)	0.704 ± 0.260	0.437 ± 0.188
Fair	199 (19.40)	0.585 ± 0.147	0.494 ± 0.141	0.647 ± 0.130	0.569 ± 0.132	110 (21.57)	0.416 ± 0.183	89 (17.25)	0.830 ± 0.183	0.537 ± 0.183
Good	386 (37.62)	0.658 ± 0.146	0.583 ± 0.141	0.728 ± 0.129	0.666 ± 0.130	173 (33.92)	0.510 ± 0.229	213 (41.28)	0.876 ± 0.156	0.626 ± 0.172
Very good	311 (30.31)	0.767 ± 0.126	0.696 ± 0.131	0.834 ± 0.100	0.776 ± 0.113	170 (33.33)	0.643 ± 0.212	141 (27.33)	0.930 ± 0.116	0.748 ± 0.152
Excellent	94 (9.16)	0.888 ± 0.107	0.831 ± 0.146	0.916 ± 0.082	0.870 ± 0.110	39 (7.65)	0.708 ± 0.232	55 (10.66)	0.957 ± 0.116	0.867 ± 0.140
*P* value*		< 0.001		< 0.001			< 0.001		< 0.001	< 0.001
Disability status										
No	812 (79.14)	0.702 ± 0.158	0.628 ± 0.163	0.777 ± 0.133	0.717 ± 0.142	405 (79.41)	0.577 ± 0.229	407 (78.88)	0.923 ± 0.108	0.698 ± 0.177
Yes	214 (20.86)	0.674 ± 0.187	0.594 ± 0.196	0.722 ± 0.168	0.653 ± 0.177	105 (20.59)	0.395 ± 0.225	109 (21.12)	0.747 ± 0.234	0.534 ± 0.215
* P* value*		< 0.001		< 0.001			< 0.001		< 0.001	< 0.001
Self-reported Socioeconomic Status – National Level (Range: 1–10)										
Low (1–4)	127 (12.38)	0.577 ± 0.173	0.489 ± 0.166	0.641 ± 0.167	0.566 ± 0.171	63 (12.35)	0.438 ± 0.248	64 (12.40)	0.820 ± 0.223	0.556 ± 0.185
Middle (5–7)	599 (58.38)	0.674 ± 0.161	0.597 ± 0.162	0.747 ± 0.138	0.683 ± 0.145	291 (57.06)	0.510 ± 0.225	308 (59.69)	0.887 ± 0.155	0.659 ± 0.190
High (8–10)	300 (29.24)	0.775 ± 0.147	0.705 ± 0.165	0.824 ± 0.125	0.765 ± 0.143	156 (30.59)	0.635 ± 0.231	144 (27.91)	0.911 ± 0.130	0.720 ± 0.198
* P* value*		< 0.001		< 0.001			< 0.001		< 0.001	< 0.001
Self-reported Socioeconomic Status – School Level (Range: 1–10)										
Low (1–4)	149 (14.52)	0.582 ± 0.178	0.485 ± 0.166	0.648 ± 0.170	0.567 ± 0.170	65 (12.75)	0.405 ± 0.234	84 (16.28)	0.780 ± 0.207	0.515 ± 0.185
Middle (5–7)	551 (53.70)	0.666 ± 0.153	0.592 ± 0.154	0.742 ± 0.136	0.679 ± 0.143	275 (53.92)	0.524 ± 0.230	276 (53.49)	0.898 ± 0.150	0.660 ± 0.177
High (8–10)	326 (31.77)	0.784 ± 0.148	0.715 ± 0.165	0.830 ± 0.120	0.772 ± 0.137	170 (33.33)	0.615 ± 0.230	156 (30.23)	0.920 ± 0.123	0.748 ± 0.190
* P* value*		< 0.001		< 0.001			< 0.001		< 0.001	< 0.001
Age (Years)	17.08 (1.37)							17.08 (1.37)		
Mean Overall Life Satisfaction Scores (Range: 0–10)	6.93 (1.94)							6.92 (2.00)		

SD, Standard Deviation; CHU9D, Child Health Utility 9D. * Kruskal-Wallis test. Psychosocial bolt-ons (Vitality, Sleep, Social Isolation, Close Relationships) are based on the four psychosocial dimensions proposed to extend the EQ-5D-5L in the empirical study by Chen and Olsen [5]

### Exploratory factor analysis

Based on the minimum average partial method, a two-factor structure emerged for LSS-Y and CHU9D, and a three-factor structure for LSS-Y and EQ-5D-5L measures. Similarly, the EFA revealed a two-factor structure for DWI with CHU9D or EQ-5D-5L, and a three-factor structure for DWI with EQ-5D-5L with psychosocial bolt-ons. Parallel analyses supported all these solutions. Detailed goodness-of-fit results are presented in Online Resource 4.

For LSS-Y and CHU9D, the two-factor solution indicated that all LSS-Y domains loaded onto Factor 1, while all CHU9D domains loaded onto Factor 2 (Table 3). For LSS-Y and EQ-5D-5L, the *anxiety* dimension of EQ-5D-5L loaded onto Factor 1 together with six LSS-Y domains (*friends*,* mental health*,* physical health*,* school*,* appearance*, and *time use*), while the remaining EQ-5D-5L domains and other LSS-Y domains loaded onto separate factors. When psychosocial bolt-ons were included, the same three-factor structure was retained, with all bolt-on dimensions loading together with the *anxiety* domain. The three-factor solutions for LSS-Y and EQ-5D-5L instruments are shown in Online Resource 4.

**Table 3:** Exploratory factor analysis comparing the Life Satisfaction Scale for Youth and CHU9D

Instruments	Items/Dimensions	Factor	
		1	2
CHU9D	Sad		0.67
CHU9D	Annoyed		0.66
CHU9D	Worried		0.60
CHU9D	Sleep		0.59
CHU9D	Tired		0.58
CHU9D	School Work		0.56
CHU9D	Daily Routines		0.52
CHU9D	Pain		0.51
CHU9D	Activities		0.39
LSS-Y	Future	0.72	
LSS-Y	Choice	0.70	
LSS-Y	Family	0.61	
LSS-Y	Appearance	0.58	
LSS-Y	Safety	0.56	
LSS-Y	Life at School	0.56	
LSS-Y	Things you have	0.54	
LSS-Y	Physical Health	0.53	
LSS-Y	Neighbourhood	0.51	
LSS-Y	Time use	0.51	
LSS-Y	Mental Health	0.49	
LSS-Y	Friends	0.42	

CHU9D, Child Health Utility 9D; LSS-Y, Life Satisfaction Scale for Youth. Extraction Method: maximum likelihood; Number of factors was determined by the minimum average partial method; Rotation Method: Oblique Promax; Root mean square residual (RMSR) = 0.046; Loadings smaller than 0.30 were suppressed; Kaiser-Meyer-Olkin (KMO) = 0.911; *p* value of the Bartlett test of sphericity = 0.000

For DWI and CHU9D, the two-factor solution showed most DWI domains loading together, while CHU9D domains loaded predominantly onto a separate factor, except for *activities* and *daily routine* dimensions, which loaded with DWI domains. For DWI and EQ-5D-5L, all DWI domains and the EQ-5D-5L *anxiety* dimension loaded onto Factor 1, whereas the remaining EQ-5D-5L dimensions loaded onto the other. In the three-factor solution including psychosocial bolt-ons, all bolt-on dimensions and the EQ-5D-5L *anxiety* dimension loaded with DWI mental health onto one factor, the remaining EQ-5D-5L domains loaded onto a second factor, and the remaining DWI domains loaded onto a third, *family*,* friendships*, and *respect and dignity* showing cross-loadings. Table 4 presents the three-factor solution for DWI and EQ-5D-5L with psychosocial bolt-ons, while the two-factor solutions for DWI with the other measures are provided in Online Resource 4.

**Table 4:** Exploratory factor analysis comparing the disability wellbeing index and EQ-5D-5L with psychosocial bolt-ons

Instruments	Items/Dimensions		Factor	
		1	2	3
EQ-5D-5L	Personal care		0.78	
EQ-5D-5L	Mobility		0.73	
EQ-5D-5L	Usual activities		0.58	
EQ-5D-5L	Pain		0.46	
EQ-5D-5L	Anxiety			0.71
Psychosocial bolt-ons	Vitality			0.68
Psychosocial bolt-ons	Social isolation			0.67
Psychosocial bolt-ons	Sleep			0.64
Psychosocial bolt-ons	Close relationships			0.61
DWI	Mental health			0.61
DWI	Housing	0.76		
DWI	Everyday activities	0.66		
DWI	Meaningful life	0.65		
DWI	Personal care	0.60		
DWI	Physical health	0.59		
DWI	Support team	0.58		
DWI	Finances	0.57		
DWI	Safety	0.57		
DWI	Learning	0.50		
DWI	Work	0.47		
DWI	Respect and dignity	0.44		0.34
DWI	Family	0.41		0.32
DWI	Friendships	0.32		0.33

DWI, Disability Wellbeing Index. Extraction Method: maximum likelihood; Number of factors was determined by the minimum average partial method; Rotation Method: Oblique Promax; Root mean square residual (RMSR) = 0.035; Loadings smaller than 0.30 were suppressed; Kaiser-Meyer-Olkin (KMO) = 0.935; *p* value of the Bartlett test of sphericity = 0.000. Psychosocial bolt-ons (Vitality, Sleep, Social Isolation, Close Relationships) are based on the four psychosocial dimensions proposed to extend the EQ-5D-5L in the empirical study by Chen and Olsen [5]

### Known-groups validity

The Kruskal–Wallis test showed statistically significant differences in the scores of the SWB and HRQoL measures across the five levels of self-reported health status (*p* < 0.001) and the three levels of self-reported SES at both national and school levels (*p* < 0.001). Across all instruments, scores increased monotonically with improving general health status. A similar gradient pattern was observed across SES categories, with lower scores among adolescents reporting low SES and higher scores among those reporting high SES. In addition, an analysis of self-reported disability status showed that both HRQoL and SWB measures significantly differentiated between adolescents with and without disability (*p* < 0.001). Table 2 presents the unadjusted summative (harmonic and arithmetic) scores for the SWB measures and utility scores for the HRQoL measures across these variables, together with the corresponding Kruskal–Wallis test results. Figures illustrating the comparisons of scores across categories of general health, SES, and disability status are presented in Online Resource 2. To examine whether these observed group differences persisted after adjustment for sociodemographic characteristics, multivariable regression analyses were conducted and are presented in “Factors associated with outcome measures” section.

### Factors associated with outcome measures

After adjustment for sociodemographic characteristics, self-reported general health demonstrated a clear and consistent gradient across all instruments. Compared with adolescents reporting excellent health, those reporting poorer health had progressively lower well-being or utility scores. This pattern was observed for both SWB and HRQoL measures, although the *very good* category was not statistically significant for CHU9D and EQ-5D-5L (See Table 5).

Associations with SES were more consistently observed for the SWB measures than for the HRQoL instruments. LSS-Y and DWI showed significant differences across both national- and school-level SES categories, whereas HRQoL measures demonstrated more limited or inconsistent associations. CHU9D showed no significant SES associations, while the EQ-5D-5L instruments detected differences primarily at the school level. At the national level, SES coefficients for EQ-5D-5L were not statistically significant, and only the high SES group was significant for CHU9D.

Disability status was consistently associated with lower HRQoL scores and lower DWI scores, whereas the association for LSS-Y was not statistically significant when harmonic mean scores were used. In sensitivity analyses using arithmetic mean scores for LSS-Y, disability status became statistically significant.

Regarding other sociodemographic characteristics, boys generally reported higher scores than girls, although differences were not statistically significant for all measures. Adolescents born outside Australia tended to report lower scores, with statistically significant associations observed for DWI and CHU9D. Age group and educational status were not significantly associated with outcomes. Table 5 presents the full regression results. Sensitivity analyses using arithmetic mean scores for the SWB measures yielded similar gradients and overall patterns (Online Resource 5).

**Table 5:** Linear regression of the scores of outcome measures on sociodemographic characteristics among Australian adolescents

	LSS-Y		DWI		CHU9D		EQ-5D-5L		EQ-5D-5L with Psychosocial Bolt-ons	
	(Harmonic mean scores)		(Harmonic mean scores)		(Utility scores)		(Utility scores)		(Utility scores)	
Variables	Coefficient	Standard errors	Coefficient	Standard errors	Coefficient	Standard errors	Coefficient	Standard errors	Coefficient	Standard errors
Self-reported General Health Status (Reference - Excellent)										
Very Good	− 0.098	(0.016) ***	− 0.069	(0.013) ***	− 0.041	(0.036)	− 0.028	(0.022)	− 0.104	(0.025) ***
Good	− 0.180	(0.016) ***	− 0.153	(0.013) ***	− 0.148	(0.037) ***	− 0.067	(0.022) **	− 0.199	(0.024) ***
Fair	− 0.240	(0.017) ***	− 0.221	(0.015) ***	− 0.213	(0.040) ***	− 0.084	(0.025) **	− 0.260	(0.027) ***
Poor	− 0.312	(0.027) ***	− 0.258	(0.023) ***	− 0.337	(0.062) ***	− 0.160	(0.039) ***	− 0.304	(0.043) ***
Self-reported SES (National Level) (Reference - Middle)										
Low	− 0.050	(0.013) ***	− 0.059	(0.011) ***	0.002	(0.029)	− 0.028	(0.020)	− 0.040	(0.022)
High	0.046	(0.010) ***	0.031	(0.008) ***	0.064	(0.021) **	0.002	(0.015)	0.011	(0.016)
Self-reported SES socioeconomic status (School Level) (Reference - Middle)										
Low	− 0.034	(0.013) **	− 0.040	(0.011) ***	− 0.039	(0.029)	− 0.065	(0.018) ***	− 0.071	(0.020) ***
High	0.060	(0.010) ***	0.037	(0.008) ***	0.020	(0.021)	0.003	(0.015)	0.030	(0.016)
Age group (Reference − 19 years)										
15 years	0.007	(0.016)	0.015	(0.014)	− 0.037	(0.036)	0.032	(0.025)	0.050	(0.027)
16 years	0.012	(0.016)	0.012	(0.013)	− 0.063	(0.034)	0.027	(0.024)	0.021	(0.026)
17 years	0.014	(0.015)	0.013	(0.013)	− 0.035	(0.033)	0.021	(0.022)	0.008	(0.024)
18 years	0.002	(0.013)	0.011	(0.011)	0.024	(0.028)	0.010	(0.020)	− 0.009	(0.022)
Gender (Reference - Girls & Others)										
Boys	0.035	(0.008) ***	0.014	(0.007)	0.046	(0.018) *	0.011	(0.012)	0.063	(0.014) ***
Born in Australia (Reference - Yes)										
No	− 0.011	(0.009)	− 0.017	(0.008) *	− 0.053	(0.021) **	− 0.010	(0.014)	− 0.002	(0.015)
Education (Reference - ≤ Secondary education)										
≥Tertiary education	0.001	(0.012)	− 0.008	(0.010)	− 0.042	(0.026)	0.021	(0.019)	0.009	(0.020)
Disability Status (Reference - No)										
Yes	− 0.017	(0.010)	− 0.032	(0.009) ***	− 0.108	(0.024) ***	− 0.153	(0.015) ***	− 0.132	(0.017) ***
Number of observations	1026		1026		510		516		516	

SES = Socioeconomic Status; Dependent variables were the harmonic mean scores of the Life Satisfaction Scale for Youth (LSS-Y) and Disability Wellbeing Index (DWI), and the utility scores of the Child Health Utility 9D (CHU9D). The harmonic mean scores were rescaled onto a 0–1 scale. Psychosocial bolt-ons (Vitality, Sleep, Social Isolation, Close Relationships) are based on the four psychosocial dimensions proposed to extend the EQ-5D-5L in the empirical study by Chen and Olsen [5]. * *p* < 0.05, ** *p* < 0.01, *** *p* < 0.001. A constant was included in the regression

## Discussions

This study compared three HRQoL and two SWB instruments among young people. The analysis contributes to the literature by examining whether these measures function as substitutes or complements in outcome evaluations, particularly for adolescents.

We found that correlations between the dimensions of the included HRQoL and SWB instruments were weak to moderate. This result aligns with previous empirical comparisons between capability well-being and HRQoL measures among adult and older populations, which reported weak to moderate correlations [17, 18]. Our study extends this evidence to adolescents, indicating limited convergence between HRQoL and SWB dimensions and reinforcing that these instruments function as complements rather than substitutes.

The psychosocial dimensions of the HRQoL instruments showed stronger correlations with the SWB measures than their physical dimensions. This pattern is consistent with a broader international evidence base indicating that HRQoL instruments incorporating psychosocial or capability-related domains demonstrate greater empirical convergence with multidimensional well-being constructs than measures focused primarily on physical health [11, 48]. Comparative studies of ICECAP and EQ-5D instruments in adult populations have similarly reported moderate correlations and only partial shared variance between capability well-being measures and health-focused instruments, with the strongest associations typically observed for psychosocial rather than physical functioning domains [17]. In adolescent populations, HRQoL instruments have also been shown to correlate more strongly with psychological and social well-being domains than with physical health dimensions [20]. Consistent with this wider literature, Chen et al. [19] compared HRQoL instruments with a capability well-being measure and reported strong to very strong correlations between psychosocial dimensions and overall well-being scores, while physical dimensions continued to show only weak to moderate associations. Similarly, de Albornoz and Chen [49] found that HRQoL instruments incorporating psychosocial dimensions demonstrated stronger correlations with SWB measures than those without such dimensions in patients with asthma. Collectively, these findings suggest that the empirical association between HRQoL and SWB measures is primarily attributable to shared psychosocial content rather than physical health domains.

The EFA results revealed limited structural convergence between the HRQoL and SWB measures in terms of latent constructs. Only the psychosocial dimension of EQ-5D-5L and its psychosocial bolt-ons loaded onto the same latent construct as conceptually similar items from the SWB measures. While SWB focuses on cognitive evaluations of life circumstances, HRQoL traditionally emphasises physical health and functioning [50, 51]. Consistent with prior research, the structural overlap between the two frameworks appears to be largely attributable to shared psychosocial content rather than physical health domains.

The EFA results presented in Table 4 further revealed that the EQ-5D-5L *anxiety* and psychosocial bolt-ons (*vitality*,* sleep*,* social isolation*, and *close relationships*) loaded strongly on a common factor together with the DWI *mental health*, suggesting that these dimensions reflect psychosocial aspects of health functioning. Although some overlap was observed with selected DWI domains that reflect close relationships and general well-being (*respect and dignity*, *family*, and *friendships*), the majority of DWI domains loaded primarily on a separate factor encompassing housing, finances, safety, learning, work, and support systems. These domains reflect broader contextual and evaluative aspects of wellbeing beyond health-related functioning. Thus, while the inclusion of psychosocial bolt-ons extends the scope of EQ-5D-5L, the measure remains primarily grounded in a health-functioning framework. In contrast, the DWI captures a wider conceptualisation of adolescent well-being, supporting the interpretation that HRQoL and SWB measures are related but conceptually distinct constructs.

For CHU9D, only capability-related dimensions loaded together with those of DWI, while none of the items overlapped with the LSS-Y factors. Taken together, these findings suggest that although HRQoL and SWB measures share some structural commonality, they capture distinct domains of adolescent well-being. Using both types of measures in combination may therefore provide a more comprehensive assessment of adolescent health and well-being.

Despite the limited agreement between HRQoL and SWB measures, all instruments demonstrated the ability to distinguish between adolescents with differing levels of self-reported general health and socioeconomic status. This finding aligns with previous evidence supporting the known-groups validity of the HRQoL instruments examined in this study. For example, multiple adolescent studies have shown that CHU9D can discriminate by general health status, although evidence regarding its sensitivity to socioeconomic differences has been less consistent [9, 34, 52]. Similarly, EQ-5D-5L has been shown to differentiate between adolescents with and without special health care needs or mental health concerns, supporting its validity with respect to general health status [53]. However, adolescent evidence on its ability to distinguish between socioeconomic groups—either in standalone form or with psychosocial bolt-ons—remains limited.

After adjustment for sociodemographic characteristics, the observed gradients by general health largely persisted, particularly for the SWB measures and the EQ-5D-5L with psychosocial bolt-ons. In contrast, differentiation between *excellent* and *very good* levels was not statistically significant for CHU9D and the EQ-5D-5L. This may reflect the stronger focus of traditional HRQoL instruments on health status and functional limitations rather than broader life evaluations [51, 54]. As such, while HRQoL measures effectively capture perceived health-related functioning, incorporating broader psychosocial or contextual domains may enhance sensitivity to more subtle variations in adolescent well-being.

The regression coefficients of SES were consistently associated with all SWB measures at both national and school levels, whereas SES effects were less consistent for the HRQoL measures and became non-significant for the EQ-5D-5L at the national level and for the CHU9D at the school level after adjustment. This pattern suggests that HRQoL instruments may have limited sensitivity to socioeconomic differences once health and related covariates are accounted for. Conceptually, this likely reflects the broader scope of SWB measures, which capture adolescents’ evaluations of life circumstances that are closely linked to material resources and perceived social position, whereas HRQoL instruments focus primarily on health states and functional limitations.

One notable finding was that disability status was not statistically significant for the LSS-Y after adjustment, although it remained significant for the DWI and all HRQoL measures. In unadjusted analyses, LSS-Y significantly differentiated between adolescents with and without disability, indicating evidence of known-groups validity at the crude level. The attenuation observed in adjusted models may reflect the close association between disability status and physical health functioning, which is more directly captured by HRQoL instruments and by disability-focused measures such as the DWI. After accounting for general health and other sociodemographic characteristics, disability status may have a more limited independent association with global life satisfaction. Together, these findings further support the interpretation that HRQoL and SWB measures capture related but distinct dimensions of adolescent well-being.

With respect to other sociodemographic factors, gender, and migration status (born in Australia or not) significantly contributed to the scores of the outcome measures, although the degree of sensitivity varied across instruments. However, age and educational status were not found as significant drivers of the health and well-being of adolescents.

There are limitations to this study. First, the data were collected via an online survey. While this approach is practical for recruiting large samples, it relies on non-probability sampling of panel members and may introduce biases such as self-selection, potentially affecting the representativeness of Australian adolescents [55]. Moreover, participation required adolescents to complete the survey independently, which may have excluded those with significant cognitive impairment, learning difficulties, or more severe disabilities who are unable to self-report and may require proxy reporting. Consequently, the psychometric findings of the included instruments may not be generalisable to the most vulnerable adolescents. Second, one of the SWB measures used in this study (LSS-Y) is a study-specific life-domain satisfaction question set rather than an established and widely validated instrument. Although it was informed by systematic review, theoretical frameworks, and adolescent consultation, its psychometric properties need further investigation. This should be considered when interpreting comparisons with established HRQoL measures. Third, the HRQoL data were collected from only half of the sample, which may have slightly limited the comparability of their coefficient results with those of the SWB measures, which were estimated using the full sample. Fourth, the dependent variables differed between the two types of measures in terms of scores (summative vs. utility scores), which restricted direct comparison of the coefficient magnitudes. Finally, because this study was undertaken exclusively in Australia, the findings may have limited generalisability to other countries. Even so, they provide important insights that can guide future cross-national comparative research.

## Conclusions

In summary, HRQoL and SWB measures included in this study capture distinct yet partially overlapping aspects of well-being in adolescents. Neither LSS-Y nor DWI showed strong correlations with any of the HRQoL instruments. This was further supported by the EFA results, which indicated limited overlap in the latent constructs between the HRQoL and SWB instruments. Collectively, these findings suggest that HRQoL and SWB instruments capture complementary dimensions of adolescent well-being.

Although all instruments were able to differentiate adolescents by general health and socioeconomic status at the unadjusted level, HRQoL measures showed reduced sensitivity to socioeconomic differences after adjustment. Patterns of association with other sociodemographic characteristics also varied across instruments, indicating that SWB and HRQoL measures respond differently to contextual and social factors.

Taken together, these findings suggest that HRQoL and SWB measures function as complementary rather than substitutable tools in adolescent outcome evaluation. Hence, they should be applied in combination within outcome evaluation studies to get a more comprehensive picture of adolescent health and well-being. Further investigation should examine which specific dimensions of HRQoL and SWB instruments contribute to the observed differences across sociodemographic groups, such as migration status. Future research should explore the development and application of preference-based scoring for SWB measures to strengthen comparability with HRQoL instruments.

## Supplementary Information

Below is the link to the electronic supplementary material.

Supplementary Material 1

## Data Availability

The data that support the findings of this study are available from authors upon reasonable request.

## References

[1] Brazier, J., Ratcliffe, J., Salomon, J., & Tsuchiya, A. (2017). Measuring and Valuing Health Benefits for Economic Evaluation (Trans. Second edition. ed. Vol.). Oxford: Oxford University Press, Incorporated. Available from: https://global.oup.com/academic/product/measuring-and-valuing-health-benefits-for-economic-evaluation-9780198725923?cc=au&lang=en&

[2] WHOQOL Group. (1995). The World Health Organization quality of life assessment (WHOQOL): Position paper from the World Health Organization. *Social Science & Medicine*, *41*(10), 1403–1409. 10.1016/0277-9536(95)00112-K 8560308

[3] Flynn, T. N., Huynh, E., Peters, T. J., Al-Janabi, H., Clemens, S., Moody, A., & Coast, J. (2015). Scoring the icecap-a capability instrument. estimation of a UK general population Tariff. *Health Economics*, *24*(3), 258–269. 10.1002/hec.3014 24254584 PMC4322472

[4] Olsen, J. A., & Misajon, R. (2020). A conceptual map of health-related quality of life dimensions: Key lessons for a new instrument. *Quality of Life Research*, *29*(3), 733–743. 10.1007/s11136-019-02341-3 31676970 PMC7028807

[5] Chen, G., & Olsen, J. A. (2020). Filling the psycho-social gap in the EQ-5D: The empirical support for four bolt-on dimensions. *Quality of Life Research*, *29*(11), 3119–3129. 10.1007/s11136-020-02576-5 32648198 PMC7591404

[6] Vik, M. H., & Carlquist, E. (2018). Measuring subjective well-being for policy purposes: The example of well-being indicators in the WHO Health 2020 framework. *Scandinavian Journal of Public Health*, *46*(2), 279–286. 10.1177/1403494817724952 28830297

[7] Hicks, S., Tinkler, L., & Allin, P. (2013). Measuring subjective well-being and its potential role in policy: Perspectives from the UK office for national statistics. *Social Indicators Research*, *114*(1), 73–86. 10.1007/s11205-013-0384-x

[8] Kwon, J., Freijser, L., Huynh, E., Howell, M., Chen, G., Khan, K., Daher, S., Roberts, N., Harrison, C., Smith, S., Devlin, N., Howard, K., Lancsar, E., Bailey, C., Craig, J., Dalziel, K., Hayes, A., Mulhern, B., Wong, G., Ratcliffe, J., & Petrou, S. (2022). Systematic review of conceptual, age, measurement and valuation considerations for generic multidimensional childhood patient-reported outcome measures. *Pharmacoeconomics*, *40*(4), 379–431. 10.1007/s40273-021-01128-0 35072935 PMC9007803

[9] Chen, G., Flynn, T., Stevens, K., Brazier, J., Huynh, E., Sawyer, M., Roberts, R., & Ratcliffe, J. (2015). Assessing the health-related quality of life of australian adolescents: An empirical comparison of the child health utility 9D and EQ-5D-Y Instruments. *Value in Health*, *18*(4), 432–438. 10.1016/j.jval.2015.02.014 26091597

[10] Winn, K. M., Woode, M. E., Aydin, G., & Chen, G. (2025). Systematic review of self-reported multidimensional instruments used to measure quality of life and subjective well-being of children and adolescents. *Social Indicators Research*, *177*(2), 671–731. 10.1007/s11205-025-03533-w

[11] Al-Janabi, H., Flynn, T. N., & Coast, J. (2012). Development of a self-report measure of capability wellbeing for adults: The ICECAP-A. *Quality Of Life Research*, *21*(1), 167–176. 10.1007/s11136-011-9927-2 21598064 PMC3254872

[12] Coast, J., Flynn, T. N., Natarajan, L., Sproston, K., Lewis, J., Louviere, J. J., & Peters, T. J. (2008). Valuing the ICECAP capability index for older people. *Social Science & Medicine*, *67*(5), 874–882. 10.1016/j.socscimed.2008.05.015 18572295

[13] Mukuria, C., Peasgood, T., McDool, E., Norman, R., Rowen, D., & Brazier, J. (2023). Valuing the EQ health and wellbeing short using time trade-off and a discrete choice experiment: A feasibility study. *Value in Health*, *26*(7), 1073–1084. 10.1016/j.jval.2023.02.008 36805577

[14] Brazier, J., Peasgood, T., Mukuria, C., Marten, O., Kreimeier, S., Luo, N., Mulhern, B., Pickard, A. S., Augustovski, F., Greiner, W., Engel, L., Belizan, M., Yang, Z., Monteiro, A., Kuharic, M., Gibbons, L., Ludwig, K., Carlton, J., Connell, J., Rand, S., Devlin, N., Jones, K., Tsuchiya, A., Lovett, R., Naidoo, B., Rowen, D., & Rejon-Parrilla, J. C. (2022). The EQ-HWB: Overview of the development of a measure of health and wellbeing and key results. *Value in Health*, *25*(4), 482–491. 10.1016/j.jval.2022.01.009 35277337

[15] Chen, G., Petrie, D., Llewellyn, G., Ratcliffe, J., Bulkeley, K., Badji, S., Woode, M. E., West, R., Howe, K., Hines, M., Olsen, J. A., Ma, H. B., Aydin, G., & Harris, A. On behalf of the Disability Wellbeing Index (DWI) Research Team. (2024). Disability wellbeing index: Items development and scoring algorithms. *Centre for Health Economics, Monash University*. 10.26180/30228757

[16] Husbands, S., Mitchell, P. M., Kinghorn, P., Byford, S., Breheny, K., Bailey, C., Anand, P., Peters, T. J., Floredin, I., & Coast, J. (2024). The development of a capability wellbeing measure in economic evaluation for children and young people aged 11–15. *Social Science And Medicine*, *360*, 117311. 10.1016/j.socscimed.2024.117311 39276395 PMC7616779

[17] Keeley, T., Coast, J., Nicholls, E., Foster, N. E., Jowett, S., & Al-Janabi, H. (2016). An analysis of the complementarity of ICECAP-A and EQ-5D-3 L in an adult population of patients with knee pain. *Health and Quality of Life Outcomes*, *14*(1), 36. 10.1186/s12955-016-0430-x 26940027 PMC4776364

[18] Davis, J. C., Liu-Ambrose, T., Richardson, C. G., & Bryan, S. (2013). A comparison of the ICECAP-O with EQ-5D in a falls prevention clinical setting: Are they complements or substitutes? *Quality of Life Research*, *22*(5), 969–977. 10.1007/s11136-012-0225-4 22723152 PMC3672090

[19] Chen, G., Ratcliffe, J., Kaambwa, B., McCaffrey, N., & Richardson, J. (2018). Empirical comparison between capability and two health-related quality of life measures. *Social Indicators Research*, *140*(1), 175–190. 10.1007/s11205-017-1788-9

[20] Ravens-Sieberer, U., Wille, N., Badia, X., Bonsel, G., Burström, K., Cavrini, G., Devlin, N., Egmar, A. C., Gusi, N., Herdman, M., Jelsma, J., Kind, P., Olivares, P. R., Scalone, L., & Greiner, W. (2010). Feasibility, reliability, and validity of the EQ-5D-Y: Results from a multinational study. *Quality Of Life Research*, *19*(6), 887–897. 10.1007/s11136-010-9649-x 20401552 PMC2892614

[21] Patton, G. C., Sawyer, S. M., Santelli, J. S., Ross, D. A., Afifi, R., Allen, N. B., Arora, M., Azzopardi, P., Baldwin, W., Bonell, C., Kakuma, R., Kennedy, E., Mahon, J., McGovern, T., Mokdad, A. H., Patel, V., Petroni, S., Reavley, N., Taiwo, K., Waldfogel, J., Wickremarathne, D., Barroso, C., Bhutta, Z., Fatusi, A. O., Mattoo, A., Diers, J., Fang, J., Ferguson, J., Ssewamala, F., & Viner, R. M. (2016). Our future: A Lancet commission on adolescent health and wellbeing. *The Lancet*, *387*(10036), 2423–2478. 10.1016/S0140-6736(16)00579-1 PMC5832967 27174304

[22] Sawyer, S. M., Afifi, R. A., Bearinger, L. H., Blakemore, S. J., Dick, B., Ezeh, A. C., & Patton, G. C. (2012). Adolescence: A foundation for future health. *Lancet*, *379*(9826), 1630–1640. 10.1016/s0140-6736(12)60072-5 22538178

[23] OECD. (2013). OECD guidelines on measuring subjective well-being. *OECD Publishing*. 10.1787/9789264191655-en 24600748

[24] Viner, R. M., Ozer, E. M., Denny, S., Marmot, M., Resnick, M., Fatusi, A., & Currie, C. (2012). Adolescence and the social determinants of health. *The Lancet*, *379*(9826), 1641–1652. 10.1016/S0140-6736(12)60149-4 22538179

[25] Blakemore, S. J., & Mills, K. L. (2014). Is adolescence a sensitive period for sociocultural processing? *Annu Rev Psychol*, *65*, 187–207. 10.1146/annurev-psych-010213-115202 24016274

[26] Easterlin, R. A. (2006). Life cycle happiness and its sources. *Journal of Economic Psychology*, *27*(4), 463–482. 10.1016/j.joep.2006.05.002

[27] McAdams, K. K., Lucas, R. E., & Donnellan, M. B. (2011). The role of domain satisfaction in explaining the paradoxical association between life satisfaction and age. *Social Indicators Research*, *109*(2), 295–303. 10.1007/s11205-011-9903-9

[28] Ratcliffe, J., Huynh, E., Chen, G., Stevens, K., Swait, J., Brazier, J., Sawyer, M., Roberts, R., & Flynn, T. (2016). Valuing the Child Health Utility 9D: Using profile case best worst scaling methods to develop a new adolescent specific scoring algorithm. *Social Science And Medicine*, *157*, 48–59. 10.1016/j.socscimed.2016.03.042 27060541

[29] Chen, G., & Olsen, J. A. (2022). How is your life? Understanding the relative importance of life domains amongst older adults, and their associations with self-perceived COVID-19 impacts. *Quality of Life Research*, *31*(8), 2281–2293. 10.1007/s11136-021-03043-5 34988850 PMC8731135

[30] Decancq, K., & Lugo, M. A. (2013). Weights in multidimensional indices of wellbeing: An overview. *Econometric Reviews*, *32*(1), 7–34. 10.1080/07474938.2012.690641

[31] UNDP (2010). Human Development Report 2010 (Trans. ed. Vol.). Available from: https://hdr.undp.org/content/human-development-report-2010

[32] Badji, S., Petrie, D., Harris, A., & Chen, G. (2026). Incorporating importance weights to measure the subjective wellbeing of people with disability? A comparison of several aggregation methods. *Social Indicators Research*, *182*(1), 11. 10.1007/s11205-025-03787-4

[33] Ratcliffe, J., Couzner, L., Flynn, T., Sawyer, M., Stevens, K., Brazier, J., & Burgess, L. (2011). Valuing child health utility 9D health states with a young adolescent sample. *Applied Health Economics and Health Policy*, *9*(1), 15–27. 10.2165/11536960-000000000-00000 21033766

[34] Ratcliffe, J., Stevens, K., Flynn, T., Brazier, J., & Sawyer, M. (2012). An assessment of the construct validity of the CHU9D in the Australian adolescent general population. *Quality of Life Research*, *21*(4), 717–725. 10.1007/s11136-011-9971-y 21837445

[35] Stevens, K., & Ratcliffe, J. (2012). Measuring and valuing health benefits for economic evaluation in adolescence: An assessment of the practicality and validity of the child health utility 9D in the Australian adolescent population. *Value in Health*, *15*(8), 1092–1099. 10.1016/j.jval.2012.07.011 23244812

[36] Herdman, M., Gudex, C., Lloyd, A., Janssen, M. F., Kind, P., Parkin, D., Bonsel, G., & Badia, X. (2011). Development and preliminary testing of the new five-level version of EQ-5D (EQ-5D-5L). *Quality of Life Research*, *20*(10), 1727–1736. 10.1007/s11136-011-9903-x 21479777 PMC3220807

[37] EuroQol Research Foundation (2019). EQ-5D-5L User Guide (Trans. ed. Vol.). Available from: https://euroqol.org/publications/user-guides

[38] Norman, R., Mulhern, B., Lancsar, E., Lorgelly, P., Ratcliffe, J., Street, D., & Viney, R. (2023). The use of a discrete choice experiment including both duration and dead for the development of an EQ-5D-5L value set for Australia. *Pharmacoeconomics*, *41*(4), 427–438. 10.1007/s40273-023-01243-0 36720793 PMC10020301

[39] Campbell, M. J., & Campbell, M. J. (2021). *Correlation and regression* (pp. 165–182). Wiley, Incorporated.

[40] Velicer, W. F. (1976). Determining the number of components from the matrix of partial correlations. *Psychometrika*, *41*(3), 321–327. 10.1007/BF02293557

[41] Zwick, W. R., & Velicer, W. F. (1982). Factors influencing four rules for determining the number of components to retain. *Multivariate Behavioral Research*, *17*(2), 253–269. 10.1207/s15327906mbr1702_5 26810950

[42] Chen, K. M. (2020). Subjective poverty, deprivation, and the subjective well-being of children and young people: A multilevel growth curve analysis in Taiwan. *Children and Youth Services Review*, *114*, 105045. 10.1016/j.childyouth.2020.105045

[43] Main, G. (2019). Child poverty and subjective well-being: The impact of children’s perceptions of fairness and involvement in intra-household sharing. *Children and Youth Services Review*, *97*, 49–58. 10.1016/j.childyouth.2017.06.031

[44] Viner, R. M., Ozer, E. M., Denny, S., Marmot, M., Resnick, M., Fatusi, A., & Currie, C. (2012). Adolescence and the social determinants of health. *Lancet*, *379*(9826), 1641–1652. 10.1016/s0140-6736(12)60149-4 22538179

[45] Goodman, E., Adler, N. E., Kawachi, I., Frazier, A. L., Huang, B., & Colditz, G. A. (2001). Adolescents’ perceptions of social status: Development and evaluation of a new indicator. *Pediatrics*, *108*(2), E31. 10.1542/peds.108.2.e31 11483841

[46] StataCorp. (2023). *Stata 18. Statistical software*. StataCorp LLC.

[47] S&P Global. (2024). *EViews 14 [Computer software]*. S&P Global.

[48] Coast, J., Smith, R., & Lorgelly, P. (2008). Should the capability approach be applied. *Health Economics? Health Economics*, *17*(6), 667–670. 10.1002/hec.1359 18457341

[49] de Albornoz, S. C., & Chen, G. (2021). Relationship between health-related quality of life and subjective wellbeing in asthma. *Journal of Psychosomatic Research*, *142*, 110356. 10.1016/j.jpsychores.2021.110356 33454566

[50] Dolan, P., Peasgood, T., & White, M. (2008). Do we really know what makes us happy? A review of the economic literature on the factors associated with subjective well-being. *Journal of Economic Psychology*, *29*(1), 94–122. 10.1016/j.joep.2007.09.001

[51] Karimi, M. (2016). J. (Ed.), Health, health-related quality of life, and quality of life: What is the difference? *Pharmacoeconomics* 34 7 645–649 10.1007/s40273-016-0389-9. 26892973

[52] Lindvall, K., Vaezghasemi, M., Feldman, I., Ivarsson, A., Stevens, K. J., & Petersen, S. (2021). Feasibility, reliability and validity of the health-related quality of life instrument Child Health Utility 9D (CHU9D) among school-aged children and adolescents in Sweden. *Health and Quality of Life Outcomes*, *19*(1), 193. 10.1186/s12955-021-01830-9 34344386 PMC8336397

[53] Reyes, N., Pan, T., Jones, R., Dalziel, K., & Devlin, N. (2025). Understanding the transition between age-specific measures of health-related quality of life: Evidence on the relationship between and comparative performance of the EQ-5D-Y-5L and EQ-5D-5L. *Value in Health*, *28*(8), 1231–1240. 10.1016/j.jval.2025.04.2161 40339642

[54] Moons, P. (2004). Why call it health-related quality of life when you mean perceived health status? *European Journal of Cardiovascular Nursing*, *3*(4), 275–277. 10.1016/j.ejcnurse.2004.09.004 15572015

[55] Matthijsse, S. M., de Leeuw, E. D., & Hox, J. J. (2015). Internet Panels, professional respondents, and data quality. *Methodology*, *11*(3), 81–88. 10.1027/1614-2241/a000094

