# Supplementary material for: Empirical comparison of health-related quality of life and subjective well-being measures in Australian adolescents
Source: Qual Life Res. 2026 Jul 4;35(8):227. doi: 10.1007/s11136-026-04331-8 (PMC13332960; doi:10.1007/s11136-026-04331-8)
Supplement: Supplementary file 1 — Supplementary Material 1 [file 11136_2026_4331_MOESM1_ESM.pdf]

Empirical Comparison of Subjective Well-Being and Health-Related Quality of Life Measures in Australian Adolescents

(Online Resources)

1 Title

Empirical Comparison of Health-Related Quality of Life and Subjective Well-Being Measures in Australian Adolescents

2 Journal Name

Quality of Life Research

3 Names of the Authors

Kaung Mon Winn[1], Kaung.Winn@monash.edu (ORCID ID: 0000-0002-4437-9409).

Maame Esi Woode[1], Maame.Woode@monash.edu (ORCID ID: 0000-0001-9138-8853).

Gang Chen[1,2,3], gangchen@unimelb.edu.au (ORCID ID: 0000-0002-8385-5965).

4 Affiliation of the Authors

[1] Centre for Health Economics, Monash University, Caulfield Campus, Caulfield East, Melbourne, Victoria 3145, Australia

[2] Cancer Health Services Research, University of Melbourne, Melbourne, Victoria 3000, Australia

[3] Peter MacCallum Cancer Centre, Melbourne, Victoria 3000, Australia

5 Corresponding Author

Kaung Mon Winn (Kaung.Winn@monash.edu)

6 Senior Author

Professor Gang Chen (gangchen@unimelb.edu.au)

Contents

Online Resource 1: Selection of Life Domains for Life Satisfaction Scale for Youth (LSS-Y) 2

Online Resource 2: Sample Selection, Characteristics of participants along with the Medians and Interquartile Ranges of Outcome Scores, and Comparisons of Instrument Scores by Disability Status 5

Online Resource 3: Spearman's Correlation between Life Satisfaction Scale for Youth Dimensions and Health-Related Quality of Life Measures Dimensions 11

Online Resource 4: Exploratory Factor Analysis 13

Online Resource 5: Linear regression of the arithmetic mean scores of subjective well-being measures on sociodemographic characteristics among Australian adolescents 18

Online Resource 1: Selection of Life Domains for Life Satisfaction Scale for Youth (LSS-Y)

Winn et al. [1] systematically reviewed self-reported multidimensional quality of life and subjective well-being measures designed for children and adolescents. This review identified the Program for International Student Assessment Well-Being Questionnaire as the instrument that most comprehensively covered the life domains commonly included in SWB measures for young populations. The questionnaire encompasses ten domains *health, appearance, what you learn at school, life at school, friends, neighbourhood, things you have, time use,* and *relationships with parents/guardians* and *teachers.* Accordingly, these domains were selected as the core attributes for the development of the LSS-Y.

Moreover, three additional life domains—*freedom of choice, future security,* and *personal safety*—were incorporated, as these are commonly included in other well-established SWB measures for young people, such as KIDSCREEN-52 [2], Multidimensional Student's Life Satisfaction Scale [3], Personal Well-being Index – School Children [4], Quality of Life Profile - Adolescent Version [5], and Youth Quality of Life instruments [6]. In addition, the *health* domain was divided into *physical health* and *mental health* to more comprehensively capture distinct aspects of health, consistent with the approach adopted in other well-being measures [7].

Using these 14 attributes, each with four response levels (*Totally satisfied, Satisfied, Not satisfied,* and *Not satisfied at all*), a questionnaire was developed to consult adolescents on the relevance of the proposed domains and the appropriateness of the wording for young people. This questionnaire was emailed to six adolescents (50% girls), aged 15–19 years, who were selected through convenience sampling. After completing the questionnaire, face-to-face, semi-structured, in-depth interviews were conducted to explore the rationale underlying participants' questionnaire responses, their views on the most relevant life domains for young people, and to refine the wording of both the domains and response levels.

Eventually, the instrument comprised 12 life domains: *physical health, mental health,* how much *choice* they have in life, what may happen to them later in their life (*future),* relationship with their *family,* the *friends* they have, *life at school* (or college/university), how *safe* they feel, *neighbourhood, time use*, the way that they look (*appearance*), and *things you have.* Cross-country empirical analyses of self-reported life domain satisfaction by Winn et al. [8] confirmed that these 12 domains are relevant and contribute meaningfully to adolescents' global life satisfaction. Each domain was evaluated using a four-point Likert scale (*Excellent, Good, Fair* and *Poor*), as suggested by the adolescents. The final LSS-Y instrument is presented below.

How satisfied are you with each of the following?

(Please select one response in each row.)

No.	Life Domains	Excellent	Good	Fair	Poor
	Your **life as a whole**	☐	☐	☐	☐
1	Your **physical health**	☐	☐	☐	☐
2	Your **mental health**	☐	☐	☐	☐
3	Your **appearance** (the way that you look)	☐	☐	☐	☐
4	Your **life at school**	☐	☐	☐	☐
5	The **friends** you have	☐	☐	☐	☐
6	The **neighbourhood** that you live in	☐	☐	☐	☐
7	All the **things that you have**	☐	☐	☐	☐
8	How you **use** your **time**	☐	☐	☐	☐
9	Your relationship with your **family**	☐	☐	☐	☐
10	What may happen to you later in your life (in the future) **[Future]**	☐	☐	☐	☐
11	How much choice you have in life **[Choice]**	☐	☐	☐	☐
12	How **safe** you feel	☐	☐	☐	☐

References

1. Winn, K. M., Woode, M. E., Aydin, G., & Chen, G. (2025). Systematic Review of Self-Reported Multidimensional Instruments Used to Measure Quality of Life and Subjective Well-Being of Children and Adolescents. *Social Indicators Research*, 177(2), 671–731. https://doi.org/10.1007/s11205-025-03533-w

2. Ravens-Sieberer, U., Gosch, A., Rajmil, L., Erhart, M., Bruil, J., Power, M., Duer, W., Auquier, P., Cloetta, B., Czemy, L., Mazur, J., Czimbalmos, A., Tountas, Y., Hagquist, C., Kilroe, J., & Group, K. (2008). The KIDSCREEN-52 quality of life measure for children and adolescents: psychometric results from a cross-cultural survey in 13 European countries. *Value Health*, 11(4), 645–658. https://doi.org/10.1111/j.1524-4733.2007.00291.x

3. Huebner, E. S. (1994). Preliminary development and validation of a multidimensional life satisfaction scale for children. *Psychological Assessment*, 6(2), 149–158. https://doi.org/10.1037/1040-3590.6.2.149

4. Cummins, R. A., & Lau, A. L. D. (2023). *Personal Wellbeing Index - School Children. 4th Edition.*

5. Raphael, D., Rukholm, E., Brown, I., Hill-Bailey, P., & Donato, E. (1996). The Quality of Life Profile--Adolescent Version: background, description, and initial validation. *J Adolesc Health*, 19(5), 366–375. https://doi.org/10.1016/S1054-139X(96)00080-8

6. aEdwards, T. C., Huebner, C. E., Connell, F. A., & Patrick, D. L. (2002). Adolescent quality of life, part I: conceptual and measurement model. *J Adolesc*, 25(3), 275–286. https://doi.org/10.1006/jado.2002.0470; bPatrick, D. L., Edwards, T. C., & Topolski, T. D. (2002). Adolescent quality of life, part II: initial validation of a new instrument. *J Adolesc*, 25(3), 287–300. https://doi.org/10.1006/jado.2002.0471

7. Chen, G., Petrie, D., Llewellyn, G., Ratcliffe, J., Bulkeley, K., Badji, S., Woode, M. E., West, R., Howe, K., Hines, M., Olsen, J. A., Ma, H. B., Aydin, G., Harris, A., & on behalf of the Disability Wellbeing Index (DWI) Research Team. (2024). *Disability Wellbeing Index - Items Development and Scoring Algorithms*: Centre for Health Economics, Monash University.

8. Winn, K. M., Chen, G., & Woode, M. E. (2025). Exploring the Complexity of the Relationship between Global Life Satisfaction and Satisfaction with Life Domains in Early Adolescents. *Child Indicators Research*. https://doi.org/10.1007/s12187-025-10288-w

Online Resource 2: Sample Selection, Characteristics of participants along with the Medians and Interquartile Ranges of Outcome Scores, and Comparisons of Instrument Scores by Disability Status

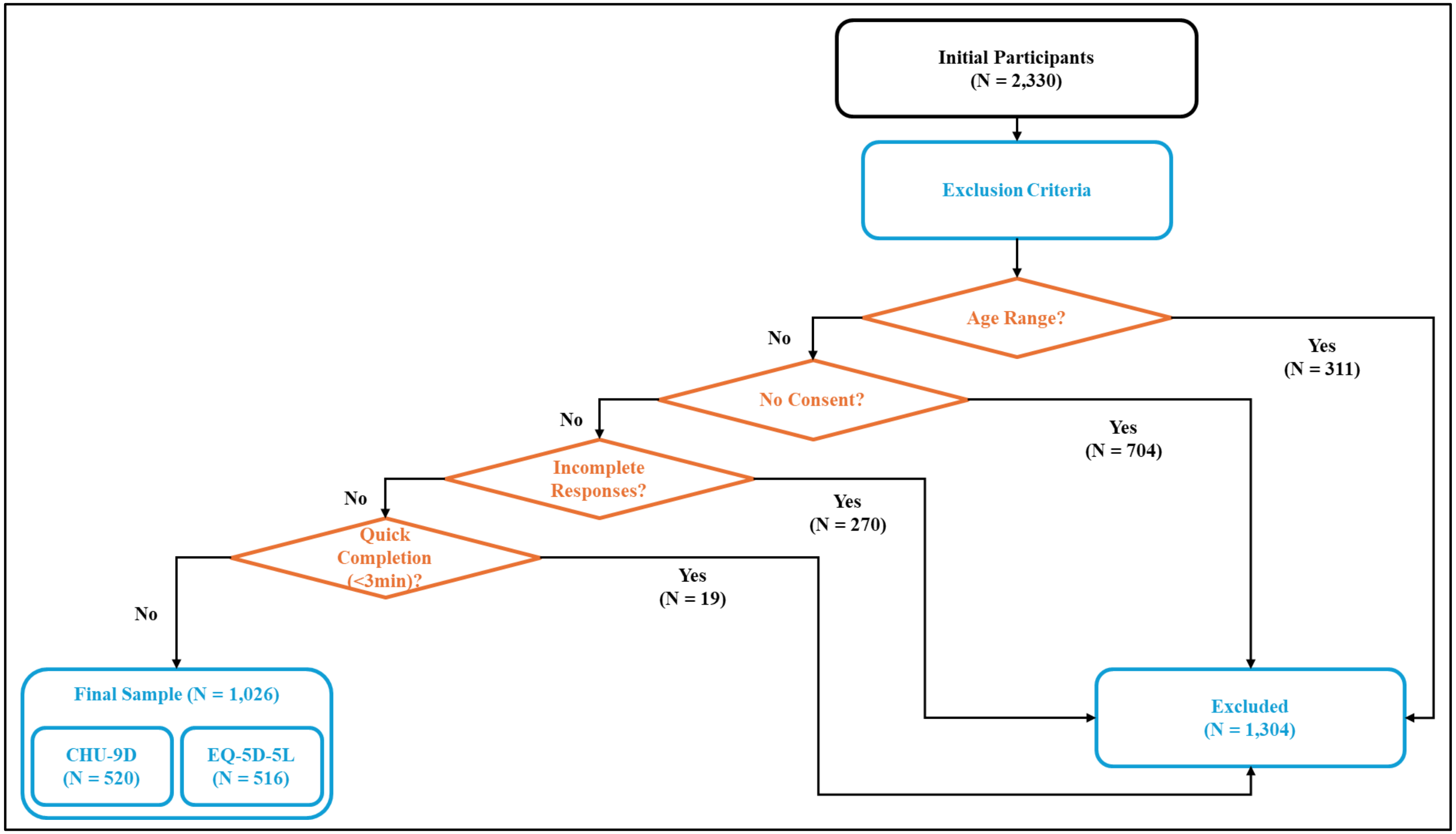

Figure A: Participant Flow to Final Sample and Subsamples

Table A: **Characteristics of participants along with the medians and interquartile ranges of outcome scores**

Participant Characteristics	Life Satisfaction Scale for Youth (LSS-Y)		Disability Wellbeing Index (DWI)		CHU9D	EQ-5D-5L	E-Q-5D-5L with Psychosocial Bolt-ons
	Harmonic Mean Scores	Arithmetic Mean Scores	Harmonic Mean Scores	Arithmetic Mean Scores	Utility Scores	Utility Scores	
	Median (IQR)	Median (IQR)	Median (IQR)	Median (IQR)	Median (IQR)	Median (IQR)	Median (IQR)
Sample Size	0.702 (0.583 - 0.812)	0.611 (0.500 - 0.722)	0.762 (0.658 - 0.868)	0.696 (0.589 - 0.804)	0.521 (0.348 - 0.719)	0.934 (0.887 - 1.000)	0.673 (0.517 - 0.820)
Age Groups							
15 years	0.718 (0.595 - 0.823)	0.639 (0.528 - 0.722)	0.775 (0.705 - 0.900)	0.732 (0.607 - 0.839)	0.502 (0.352 - 0.743)	0.956 (0.890 - 1.000)	0.502 (0.352 - 0.743)
16 years	0.718 (0.595 - 0.800)	0.639 (0.500 - 0.694)	0.766 (0.675 - 0.865)	0.696 (0.607 - 0.786)	0.462 (0.312 - 0.687)	0.934 (0.900 - 1.000)	0.462 (0.312 - 0.687)
17 years	0.693 (0.595 - 0.828)	0.611 (0.500 - 0.722)	0.767 (0.650 - 0.868)	0.714 (0.571 - 0.804)	0.547 (0.343 - 0.722)	0.934 (0.860 - 0.968)	0.547 (0.343 - 0.722)
18 years	0.685 (0.583 - 0.806)	0.611 (0.500 - 0.722)	0.762 (0.662 - 0.854)	0.696 (0.589 - 0.786)	0.566 (0.376 - 0.738)	0.934 (0.862 - 0.968)	0.566 (0.376 - 0.738)
19 years	0.685 (0.571 - 0.800)	0.611 (0.472 - 0.722)	0.756 (0.645 - 0.855)	0.679 (0.571 - 0.786)	0.528 (0.366 - 0.726)	0.924 (0.834 - 0.968)	0.528 (0.366 - 0.726)
Gender							
Girl	0.667 (0.559 - 0.762)	0.583 (0.472 - 0.667)	0.750 (0.648 - 0.846)	0.696 (0.571 - 0.768)	0.485 (0.479 - 0.753)	0.934 (0.856 - 0.968)	0.628 (0.479 - 0.753)
Boy	0.733 (0.617 - 0.853)	0.639 (0.528 - 0.750)	0.783 (0.675 - 0.893)	0.714 (0.607 - 0.821)	0.558 (0.555 - 0.866)	0.934 (0.890 - 1.000)	0.734 (0.555 - 0.866)
Unknown / Others	0.685 (0.606 - 0.788)	0.583 (0.500 - 0.667)	0.756 (0.577 - 0.838)	0.679 (0.518 - 0.768)	0.567 (0.412 - 0.826)	0.934 (0.890 - 1.000)	0.576 (0.412 - 0.826)
States							
Australian Capital Territory	0.685 (0.595 - 0.889)	0.639 (0.500 - 0.833)	0.813 (0.718 - 0.884)	0.732 (0.661 - 0.821)	0.416 (0.295 - 0.700)	0.967 (0.912 - 1.000)	0.764 (0.672 - 0.881)
South Australia	0.676 (0.533 - 0.788)	0.611 (0.472 - 0.694)	0.750 (0.623 - 0.838)	0.696 (0.571 - 0.768)	0.427 (0.336 - 0.690)	0.934 (0.887 - 0.968)	0.688 (0.494 - 0.808)
New South Wales	0.718 (0.595 - 0.823)	0.639 (0.528 - 0.722)	0.783 (0.675 - 0.882)	0.732 (0.589 - 0.804)	0.560 (0.382 - 0.722)	0.934 (0.897 - 1.000)	0.687 (0.534 - 0.844)
Tasmania	0.571 (0.444 - 0.685)	0.500 (0.361 - 0.639)	0.725 (0.641 - 0.767)	0.679 (0.536 - 0.714)	0.449 (0.236 - 0.631)	0.968 (0.756 - 1.000)	0.570 (0.388 - 0.750)
Northern Territory	0.667 (0.648 - 0.702)	0.611 (0.583 - 0.667)	0.598 (0.513 - 0.621)	0.500 (0.500 - 0.536)	0.500 (0.463 - 0.536)	0.673 (0.651 - 0.934)	0.468 (0.372 - 0.545)
Victoria	0.685 (0.583 - 0.812)	0.611 (0.500 - 0.722)	0.757 (0.650 - 0.882)	0.696 (0.589 - 0.821)	0.503 (0.351 - 0.726)	0.934 (0.834 - 0.968)	0.648 (0.492 - 0.805)
Queensland	0.685 (0.571 - 0.769)	0.597 (0.500 - 0.694)	0.741 (0.643 - 0.836)	0.661 (0.563 - 0.768)	0.485 (0.322 - 0.738)	0.934 (0.890 - 0.968)	0.649 (0.521 - 0.778)
Western Australia	0.702 (0.595 - 0.788)	0.611 (0.528 - 0.694)	0.773 (0.705 - 0.868)	0.714 (0.643 - 0.804)	0.539 (0.341 - 0.732)	0.934 (0.839 - 1.000)	0.658 (0.477 - 0.823)
Australian Born							

No	0.718 (0.617 - 0.825)	0.639 (0.528 - 0.750)	0.791 (0.682 - 0.894)	0.732 (0.607 - 0.821)	0.591 (0.430 - 0.776)	0.956 (0.903 - 1.000)	0.591 (0.430 - 0.776)
Yes	0.689 (0.571 - 0.800)	0.611 (0.500 - 0.722)	0.762 (0.655 - 0.854)	0.696 (0.571 - 0.786)	0.501 (0.331 - 0.700)	0.934 (0.876 - 0.968)	0.501 (0.331 - 0.700)
Education							
≤ Secondary education	0.702 (0.595 - 0.812)	0.611 (0.500 - 0.722)	0.767 (0.665 - 0.875)	0.714 (0.589 - 0.804)	0.516 (0.347 - 0.715)	0.934 (0.890 - 1.000)	0.686 (0.529 - 0.826)
≥ Tertiary education	0.685 (0.583 - 0.812)	0.611 (0.500 - 0.722)	0.751 (0.650 - 0.854)	0.696 (0.589 - 0.786)	0.533 (0.349 - 0.744)	0.934 (0.855 - 0.968)	0.649 (0.479 - 0.778)
General Health							
Poor	0.491 (0.344 - 0.565)	0.361 (0.278 - 0.472)	0.551 (0.451 - 0.662)	0.473 (0.393 - 0.554)	0.238 (0.119 - 0.322)	0.785 (0.565 - 0.934)	0.449 (0.322 - 0.513)
Fair	0.595 (0.476 - 0.667)	0.500 (0.389 - 0.583)	0.650 (0.555 - 0.744)	0.571 (0.482 - 0.661)	0.392 (0.297 - 0.533)	0.919 (0.738 - 0.934)	0.535 (0.410 - 0.684)
Good	0.667 (0.571 - 0.762)	0.611 (0.500 - 0.667)	0.739 (0.650 - 0.819)	0.679 (0.589 - 0.750)	0.496 (0.348 - 0.695)	0.924 (0.857 - 0.968)	0.624 (0.495 - 0.753)
Very good	0.775 (0.685 - 0.863)	0.694 (0.611 - 0.778)	0.842 (0.762 - 0.911)	0.768 (0.714 - 0.857)	0.661 (0.485 - 0.789)	0.968 (0.924 - 1.000)	0.776 (0.651 - 0.870)
Excellent	0.912 (0.817 - 0.978)	0.861 (0.722 - 0.944)	0.932 (0.875 - 0.981)	0.875 (0.804 - 0.964)	0.795 (0.521 - 0.907)	1.000 (0.956 - 1.000)	0.910 (0.775 - 1.000)
Disability Status							
No	0.702 (0.595 - 0.823)	0.639 (0.528 - 0.722)	0.791 (0.682 - 0.882)	0.732 (0.625 - 0.821)	0.559 (0.392 – 0.746)	0.956 (0.924 – 1.000)	0.711 (0.558 – 0.844)
Yes	0.685 (0.559 - 0.788)	0.611 (0.472 - 0.694)	0.731 (0.617 - 0.838)	0.661 (0.536 - 0.750)	0.354 (0.260 – 0.503)	0.823 (0.642 – 0.924)	0.492 (0.367 – 0.688)
Self-reported Socioeconomic Status – Australia (Range: 1 – 10)							
Low (1-4)	0.583 (0.476 – 0.710)	0.500 (0361 – 0.611)	0.667 (0.543 – 0.756)	0.589 (0.429 – 0.696)	0.437 (0.242 – 0.589)	0.924 (0.785 – 0.951)	0.550 (0.443 – 0.713)
Middle (5-7)	0.667 (0.583 – 0.788)	0.611 (0.500 – 0.694)	0.750 (0.653 – 0.850)	0.696 (0.589 – 0.768)	0.479 (0.323 – 0.694)	0.934 (0.886 – 1.000)	0.666 (0.515 – 0.804)
High (8-10)	0.775 (0.685 – 0.889)	0.694 (0.611 – 0.806)	0.841 (0.750 – 0.917)	0.768 (0.679 – 0.866)	0.646 (0.480 – 0.808)	0.949 (0.897 – 1.000)	0.743 (0.579 – 0.877)
Self-reported Socioeconomic Status – Community (Range: 1 – 10)							
Low (1-4)	0.606 (0.476 – 0.718)	0.500 (0361 – 0.611)	0.653 (0.555 – 0.762)	0.571 (0.464 – 0.679)	0.371 (0.250 – 0.555)	0.887 (0.604 – 0.924)	0.494 (0.379 – 0.644)
Middle (5-7)	0.667 (0.571 – 0.762)	0.611 (0.500 – 0.694)	0.750 (0.650 – 0.844)	0.696 (0.589 – 0.768)	0.503 (0.331 – 0.708)	0.934 (0.890 – 1.000)	0.667 (0.529 – 0.791)
High (8-10)	0.788 (0.685 – 0.905)	0.694 (0.611 – 0.833)	0.844 (0.750 – 0.927)	0.768 (0.679 – 0.875)	0.609 (0.432 – 0.795)	0.965 (0.924 – 1.000)	0.780 (0.617 – 0.893)

CHU9D, Child Health Utility 9D; IQR, Interquartile range

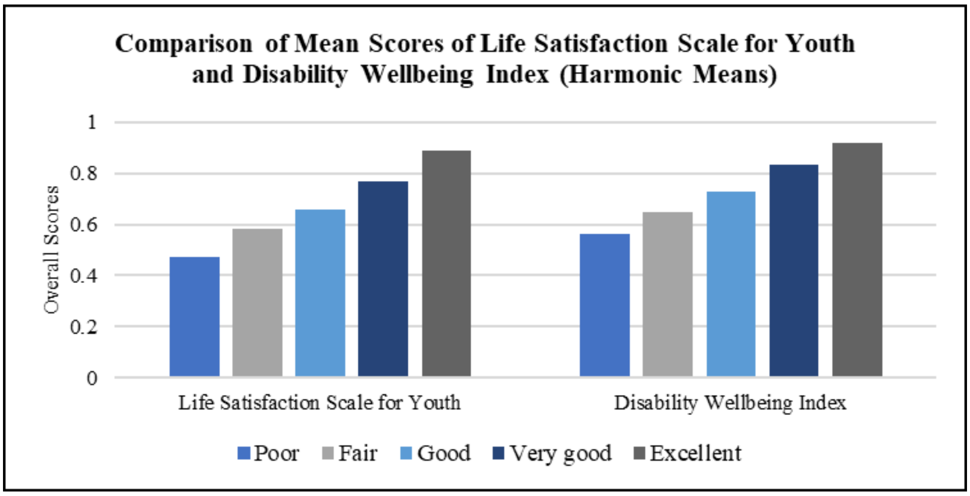
Comparison of Mean Scores of Life Satisfaction Scale for Youth and Disability Wellbeing Index (Harmonic Means)
Overall Scores
1
0.8
0.6
0.4
0.2
0
Life Satisfaction Scale for Youth
Disability Wellbeing Index
Poor
Fair
Good
Very good
Excellent

(A)

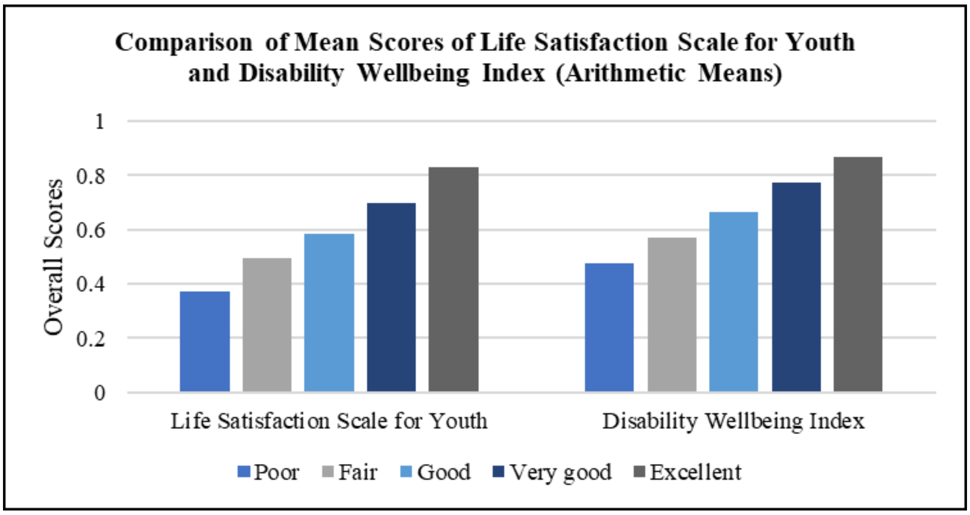
Comparison of Mean Scores of Life Satisfaction Scale for Youth and Disability Wellbeing Index (Arithmetic Means)
Overall Scores
1
0.8
0.6
0.4
0.2
0
Life Satisfaction Scale for Youth
Disability Wellbeing Index
Poor
Fair
Good
Very good
Excellent

(B)

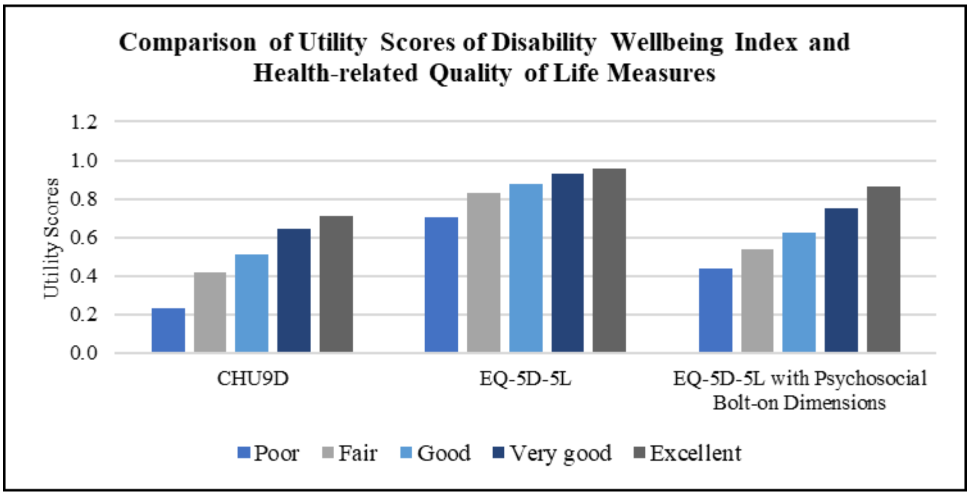
Comparison of Utility Scores of Disability Wellbeing Index and Health-related Quality of Life Measures
Utility Scores
1.2
1.0
0.8
0.6
0.4
0.2
0.0
CHU9D
EQ-5D-5L
EQ-5D-5L with Psychosocial Bolt-on Dimensions
Poor
Fair
Good
Very good
Excellent

(C)

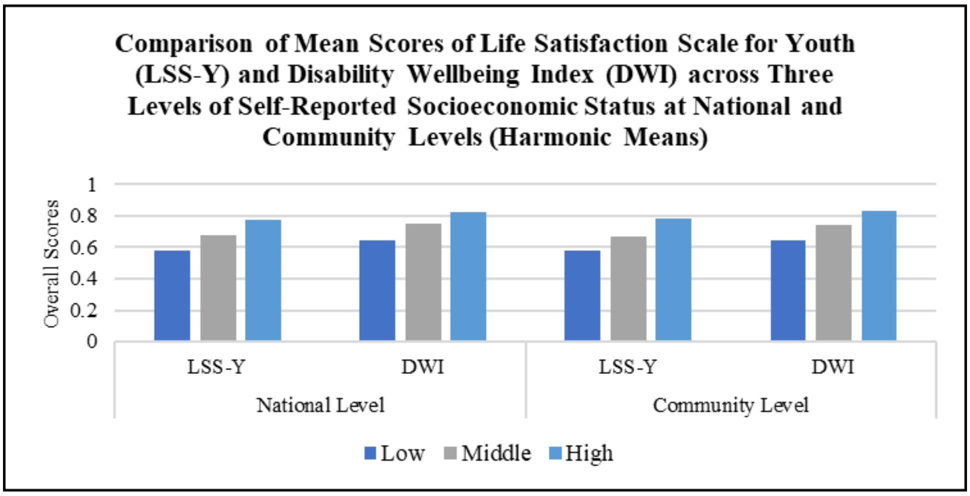

(D)

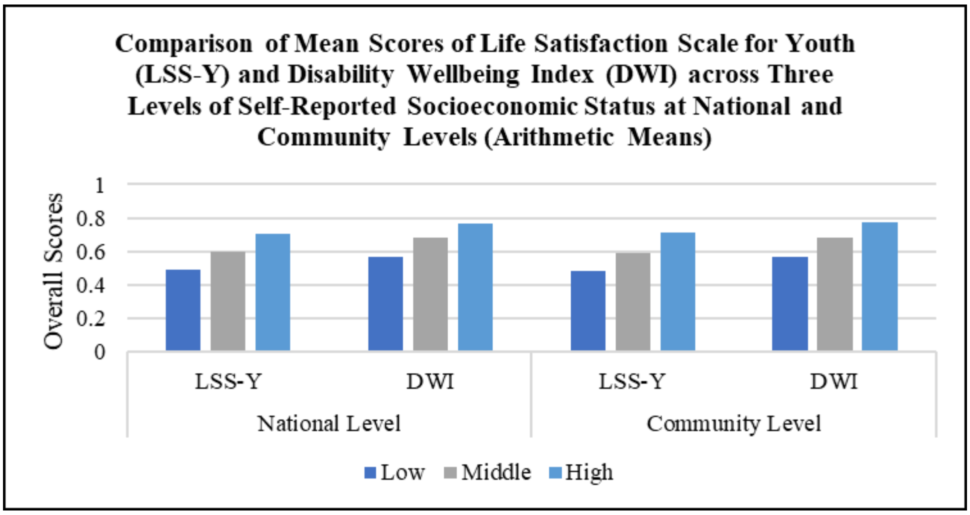

(E)

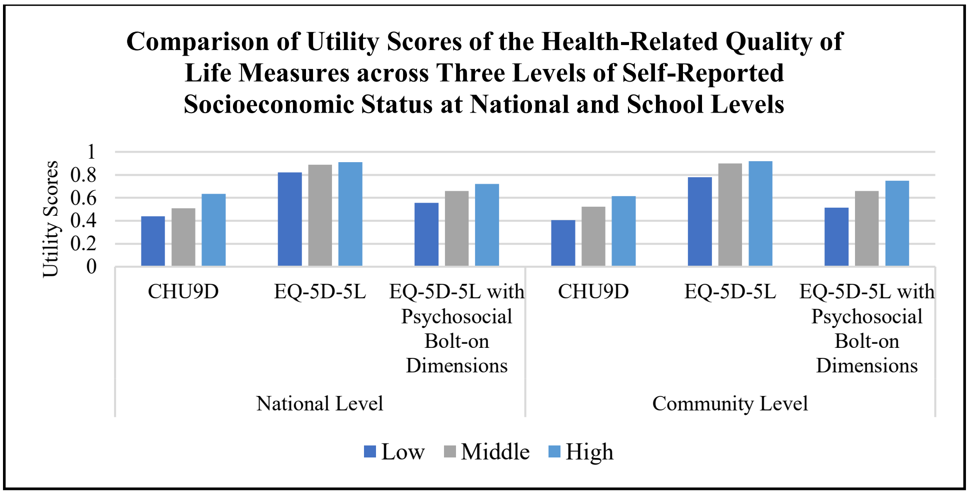

(F)

Figure B: Comparison of Mean Scores of the Well-Being Measures (A, B, D and E) and Utility Scores of the Health-related Quality of Life measures (C and F) across Five Levels of Self-Reported Health Status and Three Levels of Self-Reported Socioeconomic Status at National and School Levels

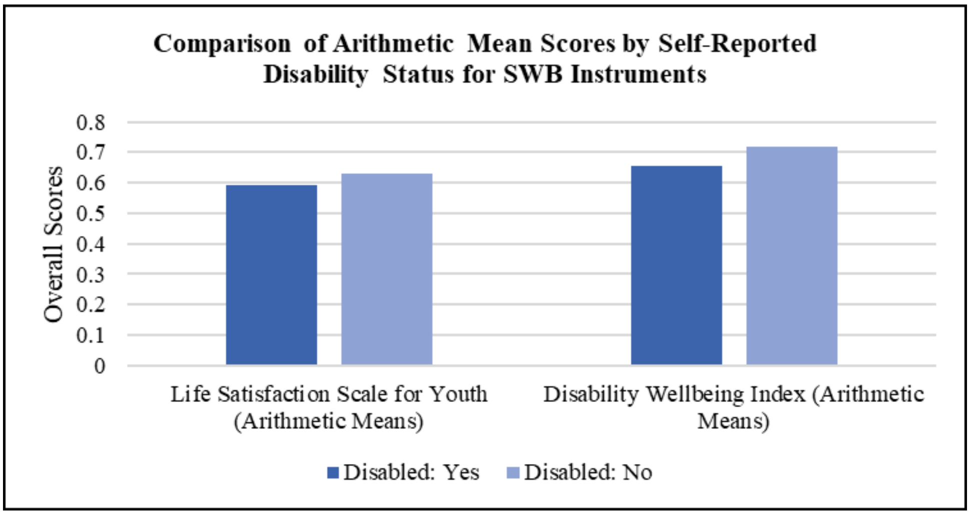

(A)

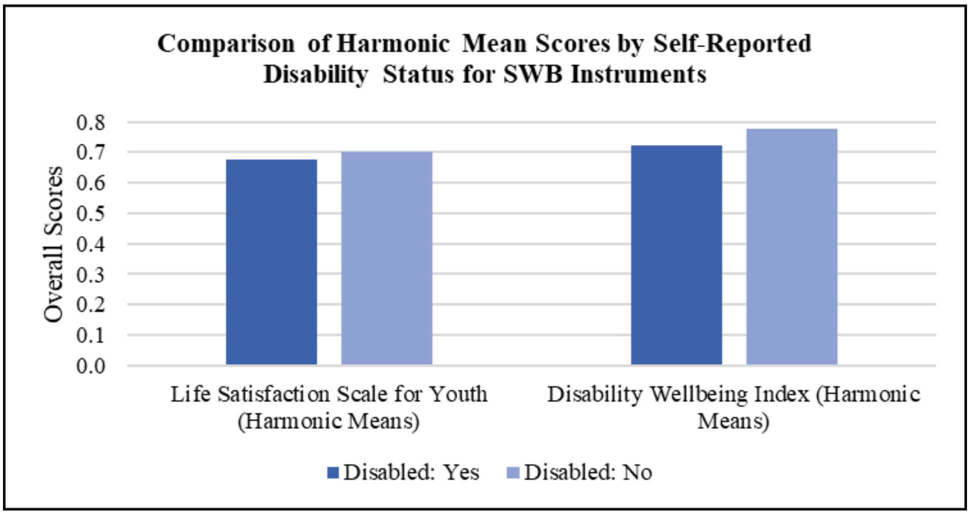

(B)

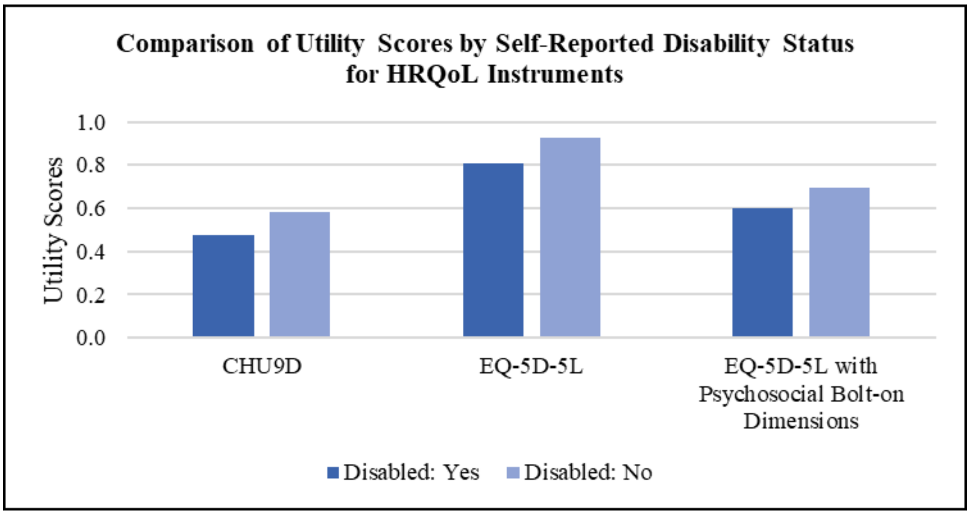

(C)

Figure C: Comparison of SWB and HRQoL Scores by Self-Reported Disability Status (SWB: A = Arithmetic Mean Scores; B = Harmonic Mean Scores; HRQoL: C = Utility Scores)

Online Resource 3: Spearman's Correlation between Life Satisfaction Scale for Youth Dimensions and Health-Related Quality of Life Measures Dimensions

Table B: Spearman's Correlation between Life Satisfaction Scale for Youth dimensions and health-related quality of life measures dimensions

	Life Satisfaction Scale for Youth											
	Physical Health	*Mental Health*	*Appearance*	*Life at School*	*Friends*	*Neighbourhood*	*Things you have*	*Time Use*	*Family*	*Future*	*Choice*	*Safety*
**CHU9D**												
Activities	0.272	0.307	0.198	0.261	0.235	0.199	0.206	0.276	0.207	0.238	0.332	0.205
Annoyed	0.200	0.289	0.162	0.161	0.193	0.176	0.245	0.210	0.241	0.173	0.228	0.227
Daily Routine	0.248	0.297	0.226	0.194	0.133	0.192	0.221	0.219	0.202	0.199	0.229	0.217
Pain	0.243	0.286	0.182	0.180	0.123	0.201	0.239	0.102	0.192	0.167	0.208	0.208
Sad	0.283	0.488	0.302	0.265	0.299	0.205	0.225	0.255	0.306	0.285	0.324	0.259
School Work	0.182	0.324	0.158	0.235	0.201	0.110	0.189	0.284	0.191	0.235	0.204	0.139
Sleep	0.196	0.283	0.164	0.136	0.157	0.139	0.153	0.223	0.151	0.205	0.219	0.188
Tired	0.319	0.381	0.212	0.297	0.149	0.166	0.147	0.342	0.159	0.232	0.215	0.163
Worried	0.260	0.377	0.253	0.293	0.257	0.102	0.155	0.325	0.222	0.256	0.275	0.223
**EQ-5D-5L**												
Anxiety	0.332	0.595	0.321	0.483	0.311	0.170	0.199	0.262	0.322	0.305	0.306	0.317
Mobility	0.065	0.070	0.064	0.075	0.068	0.128	0.123	-0.029	0.106	0.119	0.149	0.206
Pain	0.180	0.196	0.147	0.234	0.116	0.074	0.096	0.157	0.211	0.129	0.169	0.167
Personal Care	0.085	0.098	0.082	0.074	0.105	0.092	0.156	0.018	0.166	0.016	0.108	0.156
Usual Activity	0.226	0.261	0.195	0.226	0.134	0.156	0.113	0.187	0.198	0.181	0.202	0.145
**Psychosocial Bolt-on Dimensions**												
Close Relationships	0.301	0.457	0.273	0.402	0.454	0.171	0.254	0.357	0.418	0.270	0.310	0.271
Vitality	0.415	0.526	0.385	0.434	0.268	0.234	0.173	0.435	0.318	0.358	0.371	0.262
Sleep	0.301	0.437	0.229	0.299	0.229	0.153	0.195	0.284	0.276	0.240	0.259	0.236
Social Isolation	0.343	0.492	0.342	0.440	0.393	0.198	0.194	0.335	0.312	0.270	0.294	0.257

CHU9D, Child Health Utility 9D; Psychosocial bolt-ons (Vitality, Sleep, Social Isolation, Close Relationships) are based on the four psychosocial dimensions proposed to extend the EQ-5D-5L in the empirical study by Chen and Olsen [1].

Table C: Spearman's Correlation between Disability Wellbeing Index dimensions and health-related quality of life measures dimensions

	Disability Wellbeing Index													
	Family	*Friendships*	*Support Team*	*Personal Care*	*Everyday Activities*	*Meaningful Life*	*Housing*	*Physical Health*	*Mental Health*	*Learning*	*Respect and Dignity*	*Safety*	*Finances*	*Work*
**CHU9D**														
Activities	0.323	0.288	0.306	0.310	0.341	0.348	0.319	0.349	0.303	0.311	0.301	0.320	0.297	0.329
Annoyed	0.324	0.292	0.309	0.207	0.245	0.221	0.249	0.228	0.294	0.221	0.324	0.271	0.244	0.209
Daily Routine	0.240	0.210	0.243	0.430	0.389	0.311	0.305	0.295	0.309	0.260	0.240	0.292	0.311	0.290
Pain	0.219	0.128	0.184	0.239	0.241	0.202	0.256	0.230	0.276	0.194	0.252	0.286	0.328	0.233
Sad	0.417	0.322	0.257	0.274	0.294	0.317	0.341	0.320	0.467	0.306	0.348	0.323	0.323	0.303
School work	0.261	0.245	0.266	0.258	0.237	0.241	0.152	0.216	0.280	0.232	0.245	0.183	0.184	0.177
Sleep	0.216	0.223	0.238	0.276	0.309	0.270	0.240	0.231	0.243	0.235	0.268	0.291	0.245	0.286
Tired	0.257	0.212	0.177	0.209	0.211	0.245	0.176	0.302	0.333	0.233	0.261	0.211	0.259	0.260
Worried	0.362	0.322	0.230	0.238	0.297	0.306	0.286	0.337	0.413	0.255	0.283	0.285	0.246	0.274
**EQ-5D-5L**														
Anxiety	0.381	0.353	0.346	0.284	0.331	0.278	0.290	0.355	0.564	0.253	0.389	0.309	0.280	0.278
Mobility	0.086	0.194	0.183	0.243	0.176	0.139	0.230	0.184	0.083	0.168	0.184	0.232	0.121	0.049
Pain	0.212	0.160	0.251	0.231	0.232	0.174	0.194	0.281	0.204	0.209	0.211	0.254	0.201	0.134
Personal Care	0.108	0.228	0.171	0.279	0.176	0.154	0.169	0.211	0.144	0.138	0.130	0.232	0.136	0.091
Usual Activity	0.201	0.242	0.204	0.274	0.289	0.185	0.273	0.289	0.262	0.214	0.234	0.250	0.134	0.182
**Psychosocial Bolt-on Dimensions**														
Social Isolation	0.434	0.486	0.397	0.322	0.353	0.305	0.357	0.366	0.479	0.307	0.454	0.305	0.241	0.263
Sleep	0.368	0.355	0.355	0.322	0.343	0.349	0.275	0.421	0.500	0.358	0.429	0.293	0.273	0.270
Vitality	0.298	0.216	0.240	0.281	0.270	0.251	0.197	0.327	0.419	0.189	0.302	0.230	0.265	0.223
Close Relationships	0.355	0.471	0.372	0.347	0.380	0.337	0.325	0.387	0.480	0.288	0.455	0.298	0.236	0.299

CHU9D, Child Health Utility 9D; Psychosocial bolt-ons (Vitality, Sleep, Social Isolation, Close Relationships) are based on the four psychosocial dimensions proposed to extend the EQ-5D-5L in the empirical study by Chen and Olsen [1].

Reference

1. Chen, G., & Olsen, J. A. (2020). Filling the psycho-social gap in the EQ-5D: the empirical support for four bolt-on dimensions. Quality of Life Research, 29(11), 3119–3129. https://doi.org/10.1007/s11136-020-02576-5

Online Resource 4: Exploratory Factor Analysis

Table D: Goodness-of-fit summary statistics for the factor structures combining the SWB measures with the HRQoL measures

		CHU9D			EQ-5D-5L			EQ-5D-5L with Psychosocial Bolt-ons		
		2 Factor	3 Factor	4 Factor	2 Factor	3 Factor	4 Factor	2 Factor	3 Factor	4 Factor
LSS-Y	Akaike Information Criterion (AIC)	0.374	0.162	0.072	0.514	0.120	0.013	0.799	0.240	0.097
	Bayesian Information Criterion (BIC)	-1.029	-1.083	-1.024	-0.333	-0.604	-0.596	-0.592	-0.994	-0.990
	Root Mean Square Error of Approximation (RMSEA)	0.065	0.055	0.050	0.083	0.057	0.046	0.082	0.060	0.052
	Tucker-Lewis Index (TLI)	0.810	0.845	0.862	0.792	0.877	0.905	0.772	0.855	0.878
	Comparative Fit Index (CFI)	0.847	0.889	0.913	0.842	0.920	0.948	0.817	0.896	0.923
DWI	Akaike Information Criterion (AIC)	0.371	0.140	-0.037	0.372	0.146	0.039	0.779	0.185	0.047
	Bayesian Information Criterion (BIC)	-1.356	-1.413	-1.423	-0.731	-0.817	-0.792	-0.932	-1.354	-1.327
	Root Mean Square Error of Approximation (RMSEA)	0.061	0.052	0.042	0.069	0.056	0.048	0.075	0.054	0.047
	Tucker-Lewis Index (TLI)	0.829	0.860	0.889	0.846	0.881	0.901	0.797	0.871	0.889
	Comparative Fit Index (CFI)	0.859	0.896	0.927	0.879	0.919	0.942	0.833	0.904	0.927

LSS-Y: Life Satisfaction Scale for Youth; *DWI:* Disability Wellbeing Index; *CHU9D:* Child Health Utility 9D; Psychosocial bolt-ons (Vitality, Sleep, Social Isolation, Close Relationships) are based on the four psychosocial dimensions proposed to extend the EQ-5D-5L in the empirical study by Chen and Olsen [1]. Lower RMSEA, AIC, and BIC values indicate better model fit [2], whereas higher CFI and TLI values indicate superior fit relative to a null model [3]. CFI and TLI values ≥0.90 were considered acceptable and ≥0.95 excellent [4].

Reference

1. Chen, G., & Olsen, J. A. (2020). Filling the psycho-social gap in the EQ-5D: the empirical support for four bolt-on dimensions. Quality of Life Research, 29(11), 3119–3129. https://doi.org/10.1007/s11136-020-02576-5
2. Huang, P.-H. (2017). Asymptotics of AIC, BIC, and RMSEA for Model Selection in Structural Equation Modeling. Psychometrika, 82(2), 407–426. https://doi.org/10.1007/s11336-017-9572-y
3. Xia, Y., & Yang, Y. (2019). RMSEA, CFI, and TLI in structural equation modeling with ordered categorical data: The story they tell depends on the estimation methods. Behavior Research Methods, 51(1), 409–428. https://doi.org/10.3758/s13428-018-1055-2
4. Hu, L. t., & Bentler, P. M. (1999). Cutoff criteria for fit indexes in covariance structure analysis: Conventional criteria versus new alternatives. Structural Equation Modeling: A Multidisciplinary Journal, 6(1), 1–55. https://doi.org/10.1080/10705519909540118

Table E: Exploratory factor analysis comparing the Life Satisfaction Scale for Youth and EQ-5D-5L

Instruments	Items/Dimensions	Factor		
		1	2	3
EQ-5D-5L	Pain		0.50	
EQ-5D-5L	Usual Activities		0.62	
EQ-5D-5L	Mobility		0.72	
EQ-5D-5L	Personal Care		0.78	
EQ-5D-5L	Anxiety	0.63		
LSS-Y	Mental Health	0.82		
LSS-Y	Life at School	0.64		
LSS-Y	Appearance	0.60		
LSS-Y	Physical Health	0.58		
LSS-Y	Time use	0.49		
LSS-Y	Friends	0.38		
LSS-Y	Safety			0.67
LSS-Y	Things you have			0.65
LSS-Y	Choice			0.60
LSS-Y	Neighbourhood			0.48
LSS-Y	Future			0.43
LSS-Y	Family			0.39

LSS-Y – Life Satisfaction Scale for Youth; Extraction Method: maximum likelihood; Number of factors was determined by the minimum average partial method; Rotation Method: Oblique Promax; Root mean square residual (RMSR) = 0.034; Loadings smaller than 0.30 were suppressed; Kaiser-Meyer-Olkin (KMO) = 0.891; p-value of the Bartlett test of sphericity = 0.000

Table F: Exploratory factor analysis comparing the Life Satisfaction Scale for Youth and EQ-5D-5L with Psychosocial Bolt-on Dimensions

**Instruments**	**Items/Dimensions**	**Factor**		
		**1**	**2**	**3**
EQ-5D-5L	Personal Care		0.77	
EQ-5D-5L	Mobility		0.73	
EQ-5D-5L	Usual Activities		0.57	
EQ-5D-5L	Pain		0.47	
EQ-5D-5L	Anxiety	0.68		
Psychosocial Bolt-ons	Vitality	0.72		
Psychosocial Bolt-ons	Social Isolation	0.70		
Psychosocial Bolt-ons	Close Relationships	0.59		
Psychosocial Bolt-ons	Sleep	0.58		
LSS-Y	Mental Health	0.76		
LSS-Y	Life at School	0.58		
LSS-Y	Physical Health	0.50		
LSS-Y	Appearance	0.49		
LSS-Y	Time use	0.47		
LSS-Y	Friends	0.36		
LSS-Y	Things you have			0.68
LSS-Y	Safety			0.67
LSS-Y	Choice			0.61
LSS-Y	Neighbourhood			0.51
LSS-Y	Future			0.47
LSS-Y	Family			0.42

LSS-Y – Life Satisfaction Scale for Youth; Extraction Method: maximum likelihood; Number of factors was determined by the minimum average partial method; Rotation Method: Oblique Promax; Root mean square residual (RMSR) = 0.037; Loadings smaller than 0.30 were suppressed; Kaiser-Meyer-Olkin (KMO) = 0.915; p-value of the Bartlett test of sphericity = 0.000

Table G: Exploratory factor analysis comparing the Disability Wellbeing Index and Child Health Utility - 9D

**Instruments**	**Items/Dimensions**	**Factor**	
		**1**	**2**
CHU9D	Sad		0.70
CHU9D	Annoyed		0.67
CHU9D	Worried		0.60
CHU9D	Tired		0.58
CHU9D	School Work		0.54
CHU9D	Pain		0.45
CHU9D	Sleep		0.44
CHU9D	Activities	0.36	
CHU9D	Daily Routines	0.31	
DWI	Everyday Activities	0.78	
DWI	Personal Care	0.73	
DWI	Meaningful Life	0.68	
DWI	Housing	0.67	
DWI	Physical Health	0.63	
DWI	Learning	0.57	
DWI	Finances	0.55	
DWI	Work	0.52	
DWI	Mental Health	0.49	
DWI	Respect and Dignity	0.48	
DWI	Safety	0.45	
DWI	Family	0.43	
DWI	Support Team	0.41	
DWI	Friendships	0.39	

CHU9D – Child Health Utility – 9D; DWI – Disability Wellbeing Index; Extraction Method: maximum likelihood; Number of factors was determined by the minimum average partial method; Rotation Method: Oblique Promax; Root mean square residual (RMSR) = 0.046; Loadings smaller than 0.30 were suppressed; Kaiser-Meyer-Olkin (KMO) = 0.938; p-value of the Bartlett test of sphericity = 0.000

Table H: Exploratory factor analysis comparing the Disability Wellbeing Index and EQ-5D-5L

Instruments	Items/Dimensions	Factor	
		1	2
EQ-5D-5L	Personal Care		0.80
EQ-5D-5L	Mobility		0.72
EQ-5D-5L	Usual Activities		0.62
EQ-5D-5L	Pain		0.48
EQ-5D-5L	Anxiety	0.44	
DWI	Mental Health	0.70	
DWI	Meaningful Life	0.68	
DWI	Physical Health	0.68	
DWI	Respect and Dignity	0.68	
DWI	Support Team	0.68	
DWI	Housing	0.67	
DWI	Everyday Activities	0.66	
DWI	Family	0.65	
DWI	Safety	0.59	
DWI	Finances	0.58	
DWI	Personal Care	0.57	
DWI	Learning	0.56	
DWI	Work	0.56	
DWI	Friendships	0.55	

DWI – Disability Wellbeing Index; Extraction Method: maximum likelihood; Number of factors was determined by the minimum average partial method; Rotation Method: Oblique Promax; Root mean square residual (RMSR) = 0.044; Loadings smaller than 0.30 were suppressed; Kaiser-Meyer-Olkin (KMO) = 0.910; p-value of the Bartlett test of sphericity = 0.000

Online Resource 5: Linear regression of the arithmetic mean scores of subjective well-being measures on sociodemographic characteristics among Australian adolescents

	LSS-Y (Arithmetic Mean Scores)		DWI (Arithmetic Mean Scores)	
**Variables**	**Coefficient**	**Standard Errors**	**Coefficient**	**Standard Errors**
Self-reported General Health Status (Reference - Excellent)				
Very Good	-0.112	(0.015) ***	-0.081	(0.024) ***
Good	-0.194	(0.015) ***	-0.166	(0.015) ***
Fair	-0.267	(0.017) ***	-0.247	(0.014) ***
Poor	-0.339	(0.027) ***	-0.286	(0.014) ***
Self-reported SES (National Level) (Reference - Middle)				
Low	-0.056	(0.013) ***	-0.066	(0.012) ***
High	0.047	(0.010) ***	0.030	(0.009) ***
Self-reported SES socioeconomic status (School Level) (Reference - Middle)				
Low	-0.050	(0.012) ***	-0.050	(0.011) ***
High	0.060	(0.010) ***	0.039	(0.009) ***
Age group (Reference - 19 years)				
15 years	0.016	(0.016)	0.021	(0.014)
16 years	0.016	(0.015)	0.012	(0.014)
17 years	0.020	(0.015)	0.020	(0.013)
18 years	0.000	(0.013)	0.008	(0.012)
Gender (Reference - Girls & Others)				
Boys	0.038	(0.008) ***	0.015	(0.007)
Born in Australia (Reference - Yes)				
No	-0.011	(0.009)	-0.014	(0.008)

Education (Reference - ≤ Secondary education)				
≥Tertiary education	0.007	(0.012)	-0.001	(0.011)
Disability Status (Reference - No)				
Yes	-0.025	(0.010) **	-0.044	(0.009) ***
Number of Observations	1026		1026	

SES = Socioeconomic Status; Dependent variables were the harmonic mean scores of the Life Satisfaction Scale for Youth (LSS-Y) and Disability Wellbeing Index (DWI); * $p < 0.05$, ** $p < 0.01$, *** $p < 0.001$. A constant was included in the regression.
